# A Multimodal Magnetically Driven Soft Robot With Integrated Actuation‐Sensing Based on Photothermal Reprogramming Technology

**DOI:** 10.1002/advs.75817

**Published:** 2026-05-22

**Authors:** Liu Yang, Yuliao Dong, Kangning Li, Cheng Jing, Ben Wang, Lin Xu

**Affiliations:** ^1^ School of Mechanical Engineering Jiangsu University Zhenjiang P. R. China; ^2^ College of Chemistry and Environmental Engineering Shenzhen University Shenzhen P. R. China

**Keywords:** integrated actuation and sensing, magnetic soft robot, multimodal locomotion, reprogrammable magnetization, targeted drug transport

## Abstract

Conventional magnetic soft robots are fundamentally limited by static magnetization profiles and the absence of integrated perception, which restricts their adaptive functionality and environmental interactivity in dynamic settings. To address these challenges, we introduce a photothermal reprogramming strategy utilizing custom‐designed PEG/SmFeN microspheres. Within these microspheres, the intrinsic photothermal effect of SmFeN particles enables selective rewriting of magnetic domains through near‐infrared‐induced solid‐liquid phase transitions. This approach allows a single robot to achieve multiple locomotion modes and execute sophisticated shape transformations. The robot rolls substantially faster than it creeps, and the multi‐legged configuration further enhances locomotion, achieving a maximum speed of 1.8 BL/s (27 mm/s) in the multi‐legged rolling mode. Furthermore, a liquid‐metal Archimedean spiral capacitive sensor is integrated via microelectronic printing to form an actuation‐sensing system. This integrated sensor detects environmental changes and monitors self‐motion through identifiable capacitance patterns while maintaining stable operation over 100 cycles. In a simulated gastric environment, the system successfully distinguishes between rolling and creeping gaits while tracking terrain interaction, demonstrating its potential for minimally invasive medical procedures and targeted therapy. Together, these capabilities set the stage for adaptive soft robots with spatially programmable actuation, integrated multimodal sensing, and a thermally safe reprogramming profile suitable for biomedical use.

## Graphical abstract

This work reports a thermally safe, photothermally reprogrammable magnetic soft robot with its reprogramming temperature tuned to ∼39.5°C for biocompatibility. It achieves multimodal locomotion (max speed of 1.8 BL/s) and integrates liquid‐metal capacitive sensing to monitor self‐motion and detect environmental changes in simulated gastric environments, demonstrating potential for minimally invasive biomedical applications.

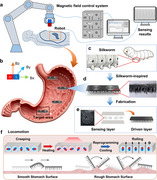

## 1 Introduction

Compared with traditional rigid robots, soft robots mostly use soft elastic materials capable of withstanding large strains as actuation media, abandoning hard materials, rigid structures, complex sensing‐control systems, and mechanical joints of traditional rigid robots [1]. They can significantly adjust morphological structures, stiffness, and motion modes over a wide range. Owing to their high flexibility, environmental adaptability, and biocompatibility, soft robots exhibit great potential in medicine, detection, and rescue [2, 3, 4, 5, 6]. Their actuation methods mainly include pneumatic/hydraulic actuation [7, 8, 9], electromagnetic actuation [10, 11, 12], light‐driven actuation [13, 14], and chemically driven actuation [15, 16, 17]. Among these, magnetically controlled soft robots [18, 19, 20] possess unparalleled application advantages in confined spaces, benefiting from the high magnetic penetrability and remote actuation‐control capabilities. With remote manipulation, rapid actuation, programmability, and inherent safety, they can non‐invasively enter narrow spaces such as blood vessels, esophaguses, and body cavities for complex tasks [21, 22], demonstrating unique potential in targeted drug delivery [23, 24, 25, 26], micro‐assembly [27, 28], minimally invasive surgery [29, 30, 31], and cell manipulation. Recent advances in magnetically controlled microrobots have demonstrated remarkable progress in wireless actuation and biomedical functionality. For instance, Xu's team proposed a multi‐level vascular embolization strategy using temperature‐magnetic dual‐responsive microrobots delivered via catheter, enabling flexible navigation into regions beyond catheter reach for complex embolization [25]. Wang et al. developed a magnetically driven blood hydrogel fiber robot using patient‐derived blood. Inspired by nematode locomotion, this robot achieves multimodal biomimetic movements for precise, minimally invasive drug delivery to deep intracranial regions via cerebrospinal fluid pathways [32]. Additionally, Xu's team developed a flexible magnetic localization patch inspired by A‐GPS, employing a dual‐stage positioning paradigm for radiation‐free, high‐precision tracking of in vivo devices across vascular and gastrointestinal interventions [33]. Despite these significant contributions, the practical applications of current magnetic soft robots are severely limited by two fundamental challenges. First, current magnetically controlled soft robots have actuation behaviors restricted by pre‐programmed internal magnetic domain orientations. Once the magnetic domains are fixed via programming, they cannot be reprogrammed, limiting flexible application in complex environments and compromising their functional versatility. Second, and equally critically, most existing systems lack integrated sensing capabilities, rendering them unable to perceive and understand their surroundings in real‐time, such as discerning contact objects or physical variations like gastric peristalsis and surface topography. This sensory blindness hampers their ability to make autonomous behavioral adjustments based on environmental feedback. Therefore, breaking the constraints of fixed actuation modes and endowing these robots with environmental perception intelligence has become one of the most critical and urgent scientific challenges for advancing magnetic soft robots toward true autonomy and clinical utility.

Currently, magnetic reprogramming of magnetically controlled soft robots mainly relies on two strategies: resetting magnetization by heating embedded magnetic particles above Curie temperature, or achieving mechanical rotational orientation of magnetic particles while maintaining constant magnetization. The former is often based on materials with low Curie temperatures (e.g., CrO_2_). For instance, Team Sitti M from Max Planck Institute for Intelligent Systems (Germany) proposed a thermally assisted magnetic programming strategy [34], which heats the magnetic soft materials above the Curie temperature of embedded ferromagnetic particles [35] and applies an external magnetic field during cooling to reorient magnetic domains. However, this method requires high‐temperature treatment (above 100°C), imposing stringent experimental conditions. The latter typically involves encapsulating ferromagnetic particles with low‐melting‐point polymers or alloys [36, 37], or using dynamic polymer matrices to achieve physical rotation of magnetic particles. Both methods require external heat sources (e.g., Joule heaters or near‐infrared light) to bring the system into a programmable state, followed by remagnetization or directional magnetic field application for reconfiguration. Nevertheless, existing methods’ reliance on harsh external conditions (high temperature, high magnetic field intensity) severely limits applicability in mild, safe‐required environments (in vivo operations, precision medical devices). Thus, developing a new method for high‐precision, reversible magnetic reconfiguration under mild conditions has become a key challenge for advancing practical application of magnetically controlled soft robots.

**FIGURE 1:**
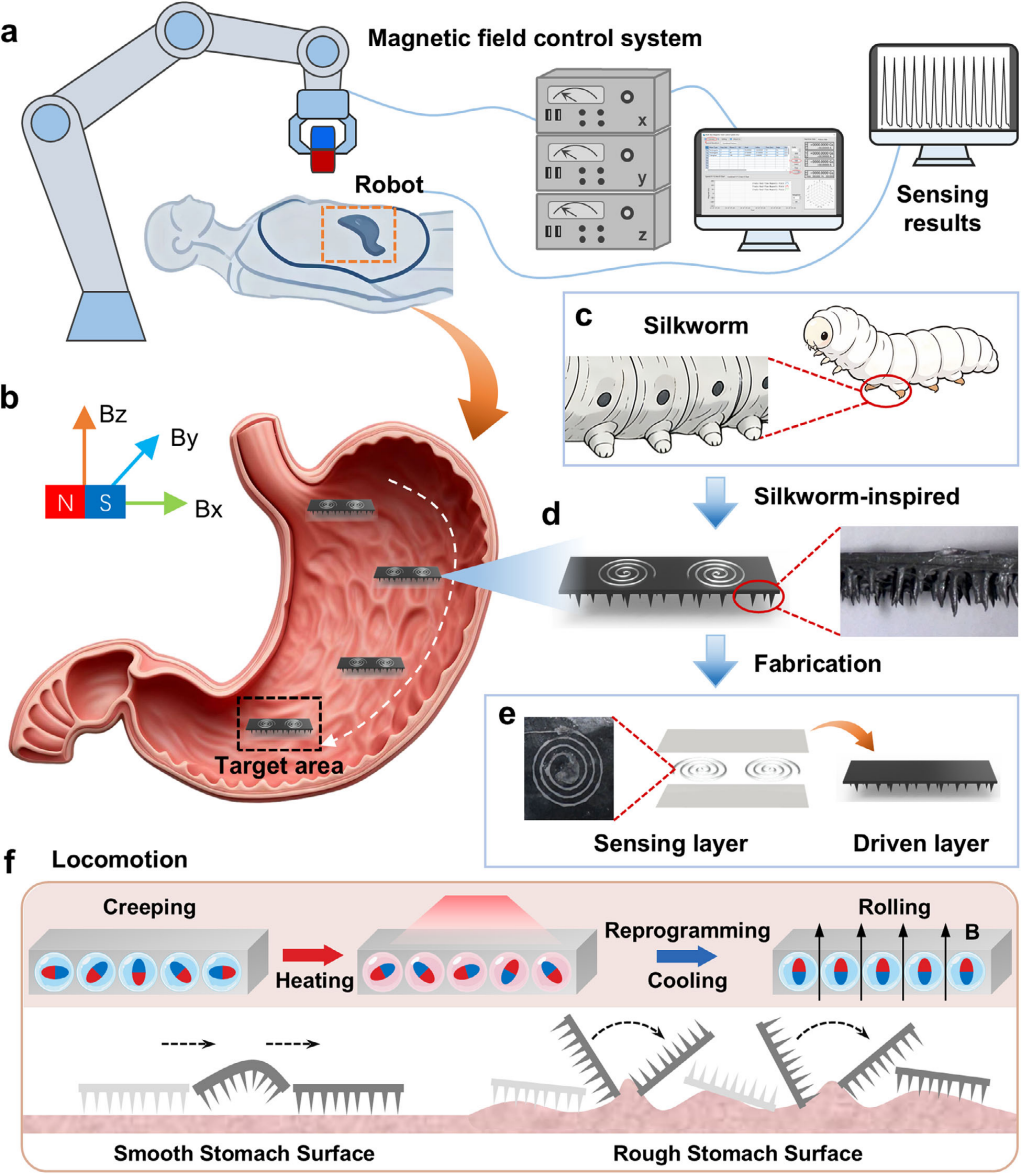
(a) Schematic showing the robot's magnetic drive system and sensing system. (b) Schematic showing intragastric locomotion of a multi‐legged sensing robot. (c) The multi‐legged bionic prototype of the robot is modeled after the abdominal legs of a silkworm. (d) Detailed view of the robot's conical multipods. (e) The robot's driven layer and sensing layer. (f) Different locomotion modes of the robot before and after reprogramming: switching from creeping to rolling.

To address these dual challenges, this study designs a functionally reconfigurable magnetically driven soft robot with integrated perception capabilities based on PEG/SmFeN/PDMS composite material. By adopting low‐temperature heating‐based reprogrammable magnetization technology, the solid‐liquid phase transition of PEG‐SmFeN magnetic microspheres induced by infrared irradiation is utilized to regulate the magnetic domain structure of the microspheres, thereby achieving the tunable response of the robot. Simultaneously, we integrate a liquid metal capacitive sensor via microelectronic printing to provide real‐time environmental and self‐motion sensing. In addition, 4D printing [38] technology is used for robot fabrication, with initial magnetic programming and curing during printing to achieve structure‐function synergistic design. To verify the feasibility of this technology, multiple prototypes of magnetically driven soft robots are constructed, and deformation/actuation performance before and after reprogramming is comparatively analyzed. Finally, a magnetically driven soft robot with a conical multi‐legged structure is designed, embodying the integrated actuation‐sensing system, integrated with a liquid metal capacitive sensor [39], and applied in a gastric model for locomotion and sensing experiments (Figure 1). This work is expected to lay a foundation for the further application of intelligent and adaptive magnetically driven soft robots in the medical field.

## 2 Results and Discussion

### 2.1 Reprogramming Strategy and Mechanism

The field of magnetic soft robotics faces a persistent challenge: the irreversible encoding of magnetic domains restricts each robot to a single, predetermined function, severely limiting its adaptability in dynamic environments. Addressing this limitation requires breakthrough technology for post‐fabrication magnetic reconfiguration. Here we present a transformative strategy based on near‐infrared light‐triggered solid‐liquid phase transitions in custom‐synthesized PEG/SmFeN microspheres (Figure S1). This methodology enables dynamic rewriting of magnetic domain patterns in PEG/SmFeN/PDMS magnetoelastic composites, establishing a new class of photothermal‐magnetic reprogrammable materials. Our work demonstrates a low‐temperature photothermal reprogramming platform that circumvents the constraints of existing magnetic domain engineering approaches. The printing platform, as shown in Figure S2, consists of both hardware and software systems.

**FIGURE 2:**
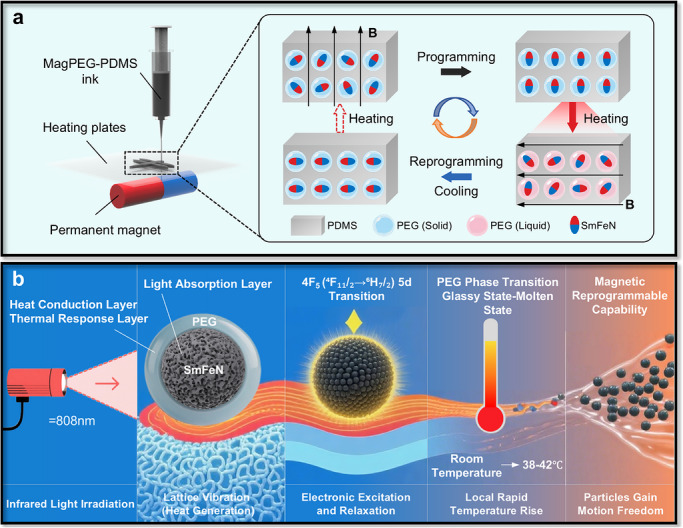
(a) Mechanism of magnetic reconfiguration. (b) The photothermal effect principle of reconfigurable magnetic materials.

As schematically illustrated in Figure 2b, under targeted near‐infrared (808 nm) illumination, SmFeN particles within the PEG/SmFeN microspheres generate localized thermal energy through efficient photothermal conversion. This heating elevates the temperature of the surrounding PEG matrix above its melting transition, inducing precisely controlled solid‐to‐liquid phase change. Within these liquefied microdomains, the previously immobilized SmFeN particles acquire rotational freedom, permitting their physical reorientation under application of relatively weak external magnetic fields. Cessation of illumination triggers rapid solidification of the PEG microspheres, permanently fixing the new magnetic domain configuration within the PDMS matrix (Figure 2a). Importantly, this reprogramming strategy enables both global and local magnetic domain reconfiguration, as demonstrated in Figure S3. This distinctive photothermal melting‐magnetic reorientation‐solidification locking cycle exhibits exceptional reproducibility through multiple cycles (Figure S4a,b and Movie S1), providing a reliable foundation for dynamic robot reconfiguration. Comprehensive experimental characterization verifies that the material system maintains stable solid‐liquid‐solid phase transition cycles across varying NIR intensities, demonstrating remarkable cyclic stability and reprogramming reliability. Although all reprogramming experiments in this work are performed ex vivo (in a simulated gastric environment), the ultimate goal is to enable in situ reprogramming in future biomedical applications. To address the thermal safety concern for in vivo applications, we have optimized the phase‐change matrix. For subsequent in vivo applications, the 1:2 blend of PEG1000:PEG1500 (melting point ∼39.5°C) can be selected because it is slightly above body temperature (37.5°C) but remains well below the thermal injury threshold (42°C), providing a sufficient safety margin.

To further evaluate the spatial temperature distribution and thermal safety of the robot during NIR irradiation, infrared thermal imaging was performed to monitor the temperature evolution of different regions on the magnetic robot and its surrounding environment (Figure S4c,d). Three representative regions were selected: Region A (core/heating zone, the highest temperature point), Region B (outer surface/limb), and Region C (adjacent ambient area, simulating tissue contact). As shown in Figure S4d, a clear temperature gradient was observed: the core temperature (A) rose rapidly to ∼49°C within 11 s, while the outer surface temperature (B) increased moderately to ∼42°C, sufficient to trigger the solid‐liquid phase transition of PEG for magnetic reprogramming, and the surrounding contact temperature (C) remained stable at ∼27°C with negligible heat diffusion. Notably, the outer surface temperature was maintained within the clinical thermal safety threshold (<42°C), while minimal thermal spread was observed under the present experimental conditions, demonstrating a notable thermal safety margin. These results confirm that the robot's core can be reprogrammed without overheating the surface or harming surrounding tissue, as a result of the localized photothermal conversion of SmFeN particles and the thermal barrier effect of the polymer matrix. Thus, for a future in situ scenario, the surrounding tissue is protected by the combination of a low phase‑change temperature (∼39.5°C) that is only slightly above body temperature, highly localized photothermal heating confined to the robot's core, and the thermal barrier property of the PDMS matrix.

### 2.2 Versatile Robotic Reconfiguration and Morphing

**FIGURE 3:**
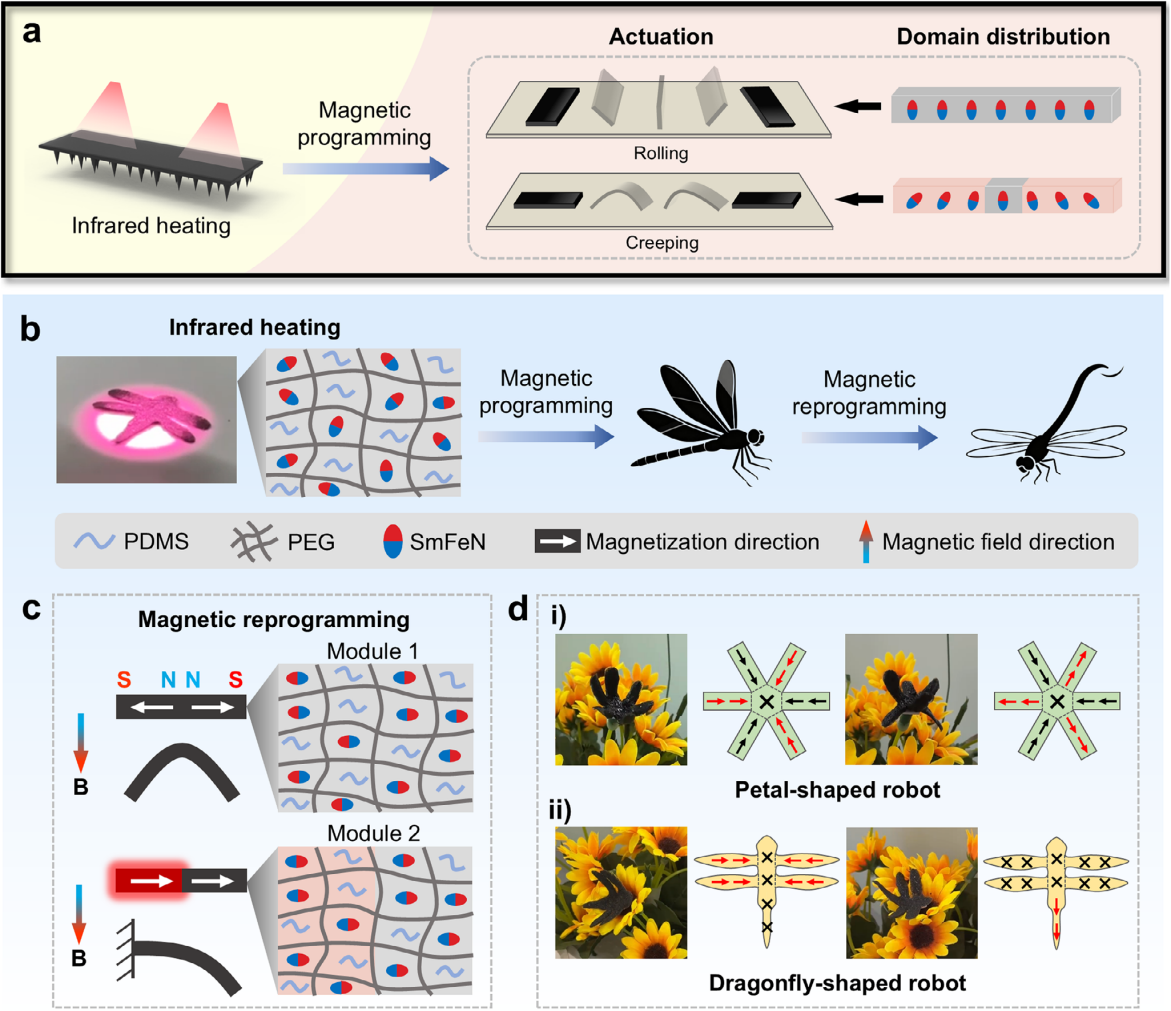
(a) Multimodal locomotion of the robot under magnetic reprogramming. (b) Initial magnetic domain arrangement and corresponding segment‐specific deformation of the dragonfly robot after reprogramming. (c) Distinct shape morphing and internal magnetic domain distributions achieved through two programming modes. (d) Comparison of deformation behaviors of bioinspired robots (petal, dragonfly structures) before and after reprogramming.

The paradigm‐shifting capability of our approach emerges from its successful decoupling of robotic structure from function. Through spatially selective photomagnetic programming of PEG/SmFeN/PDMS composites, individual robotic structures exhibit diverse behavioral modalities: a strip‐shaped robot demonstrates different locomotion patterns including rolling and creeping (Figure 3a and Figure S5), a single floral structure achieves reversible morphological transitions between closed and bloomed configurations (Figure 3d i and Movie S2), while a monolithic dragonfly robot executes independent wing and tail motions (Figure 3d ii and Movie S3). Similarly, the palm‐shaped robot can achieve 1–6 different hand gesture transformations through reprogramming (Figure S6 and Movie S4). The arrangement of magnetic domains inside the robot before and after reprogramming is shown in Figure 3b,c. This capacity for spatiotemporally selective magnetic domain reprogramming within a unified material architecture represents a fundamental advance beyond current technological capabilities. The integration of bespoke material design with sophisticated reprogramming mechanisms revealed in this work opens new design paradigms for soft robotics. The established platform lays the groundwork for developing genuinely environmentally adaptive intelligent systems, with particular promise for biomedical applications, including targeted therapy and minimally invasive surgery.

### 2.3 Material Characterization and Actuation Performance Analysis

**FIGURE 4:**
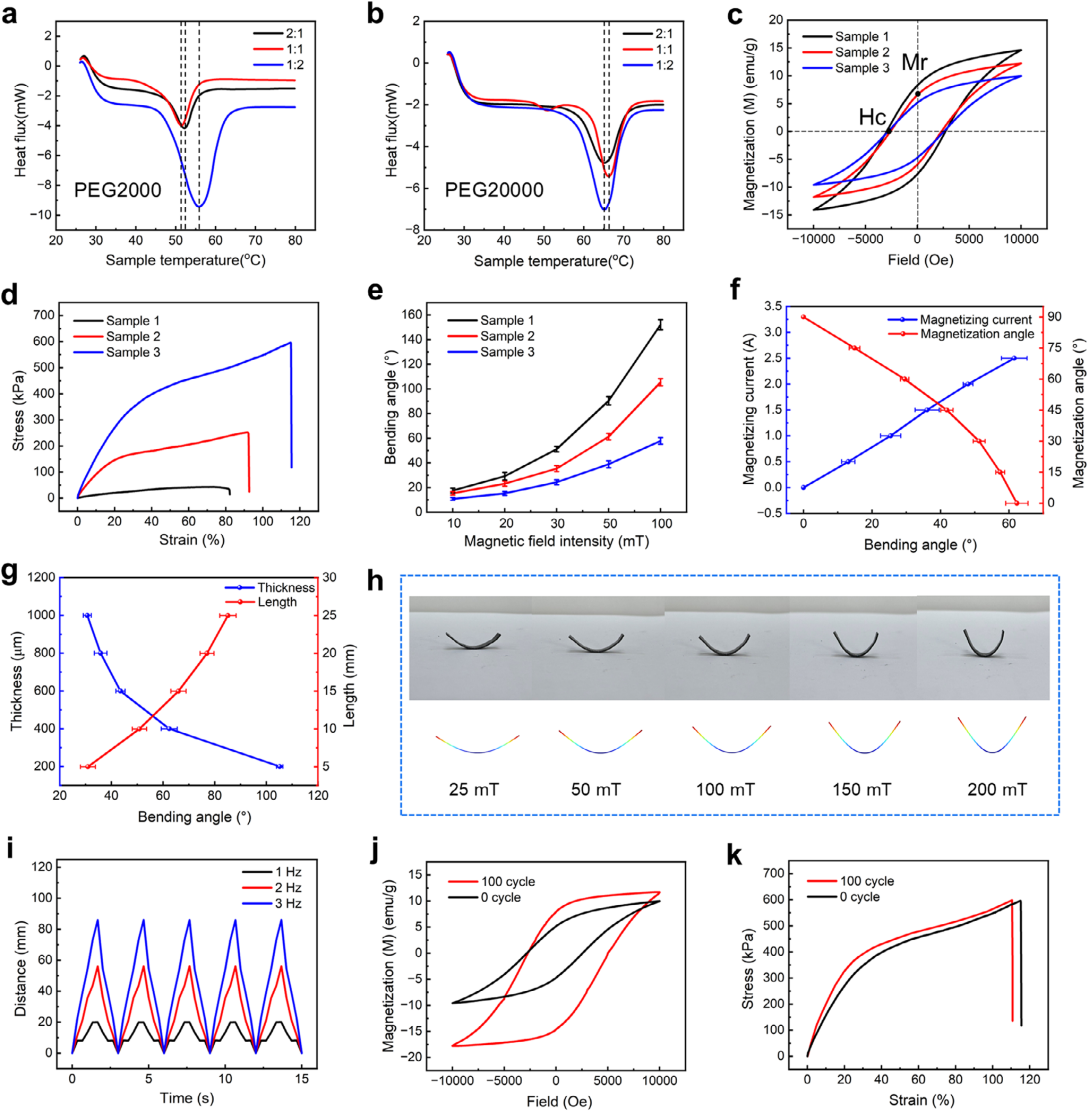
(a) DSC thermograms of PEG2000 composites at varied mass ratios. (b) DSC thermograms of PEG20000 composites at varied mass ratios. (c) Magnetic hysteresis loops of PEG/SmFeN/PDMS composites with different mass ratios. (d) Stress–strain curves of composites with different magnetic microsphere to PDMS ratios. (e) Bending angles under varying magnetic field intensities (5 mm × 15 mm samples). (f) Bending angles under different magnetizing currents and magnetization angles. (g) Bending angle vs. thickness/length under a 100 mT magnetic field. (h) Simulated deformation under different magnetic field intensities. (i) Motion response under different magnetic field frequencies (5 mm × 15 mm). (j) Magnetic properties before and after 100 heating‑reprogramming cycles. (k) Mechanical properties (stress–strain curves) of the magnetic material before and after 100 heating‑reprogramming cycles.

DSC analysis of magnetic microspheres identifies distinct phase behaviors governed by SmFeN:PEG ratio and molecular weight. As shown in Figure 4a, PEG2000 systems exhibit sharp melting endotherms at 50°C for the 2:1 and 1:1 ratios, while the 1:2 formulation shows peak broadening, suggesting higher energy barriers with excess PEG. In contrast, PEG20000 systems (Figure 4b) display elevated transition temperatures (65°C) and marked hysteresis, consistent with chain entanglement restricting molecular mobility. The superior kinetics and lower energy barrier of the PEG2000 1:1 composite establish it as the optimal choice for efficient magnetic reprogramming. The phase transition temperature of PEG is highly dependent on its molecular weight: PEG1000 exhibits a melting range of 35°C–40°C, PEG1500 43°C–48°C, and PEG2000 48°C–54°C. To further lower the reprogramming temperature for biomedical safety, we investigated blends of PEG1000 and PEG1500. The DSC results (Figure S7) show that the melting points of the 2:1, 1:1, and 1:2 blends are approximately 41.2°C, 44.8°C, and 39.5°C, respectively. Among them, the 1:2 blend exhibits the lowest melting point (39.5°C) even though it contains a higher proportion of the higher‑melting component (PEG1500). This phenomenon is attributed to a eutectic‐like interaction between the two PEG components: the two molecular chains mutually interfere with crystallization, resulting in thinner and less perfect crystallites, which depress the melting point. Therefore, the 1:2 blend can be selected as the phase‑change matrix for subsequent in vivo applications.

VSM measurements [40, 41, 42] of PEG/SmFeN/PDMS composites with varying mass ratios (4:3, 1:1, 3:4, designated Samples 1–3) confirm their hard magnetic characteristics (Figure 4c). Under a 10 kOe field, saturation magnetization (M_s_) values reach 14.61, 12.24, and 9.97 emu/g for Samples 1–3, respectively, with substantial remanence (M_r_) of 8.26, 6.53, and 5.15 emu/g persisting after field removal. This significant remanence enables sustained actuation in low‐field environments, crucial for biomedical applications. All samples maintain a consistent coercivity (Hc = 2.52 kOe) and broad hysteresis loops, ensuring magnetic stability for precise motion control. These magnetic properties directly govern the actuation performance, as evidenced by the progressive enhancement in bending deformation with increasing magnetization values under identical field conditions (Figure 4h).

Uniaxial tensile testing reveals distinct composition‐mechanical property relationships in PEG/SmFeN/PDMS composites [43, 44, 45] (Figure 4d). Sample 3 demonstrates optimal performance with a tensile strength of approximately 600 kPa, a fracture elongation of about 120%, and the highest modulus, indicating a superior strength‐toughness balance from uniform SmFeN dispersion. This microstructure reinforces stiffness while preserving matrix deformability. Conversely, Samples 2 and 1 show progressively inferior properties (∼250 kPa/90% elongation and ∼50 kPa/80% elongation, respectively) due to increased particle agglomeration.

SEM analysis reveals significant microstructural evolution in PEG/SmFeN/PDMS composites with decreasing magnetic microsphere content (Figure S8a). Samples 1 and 2 (ratios 4:3 and 1:1) show rough surfaces with pronounced particle agglomeration, indicating insufficient dispersion and heterogeneous regions. In contrast, Sample 3 (3:4 ratio) exhibits uniform surface topography and homogeneous particle distribution, reflecting enhanced interface compatibility. These structural advantages explain Sample 3's superior magnetic response uniformity and mechanical integrity, as uniform dispersion minimizes magnetic domain distortion and reduces interfacial stress concentration, thereby validating the 3:4 ratio as the optimal composition.

Magnetic field‐dependent bending characterization reveals systematic actuation trends across compositional and geometric parameters. All samples exhibit increasing bending angles with magnetic field intensity, where Sample 1 deforms significantly more than Sample 3 under identical conditions, confirming its enhanced magneto‐mechanical sensitivity from higher magnetic particle content (Figure 4e). Under a 100 mT field, bending is synergistically controlled by both field strength and domain orientation: increased magnetizing current strengthens domain deflection, while a reduced magnetization angle amplifies bending via magnetic torque enhancement (τ∝*sin*θ, Figure 4f). Geometric analysis further shows that under 100 mT excitation, bending angle decreases with increasing strip thickness but increases with increasing length, confirming the coupled influence of dimensional parameters (Figure 4g).

Frequency‐response analysis of the 3:4 composite strip (5 mm × 15 mm) shows that increasing field frequency from 1 to 3 Hz substantially enhances displacement per cycle (Figure 4i and Movie S5). This improvement stems from shorter cycle duration and improved synchronization between magnetic switching and stress relaxation, revealing a coupled “field frequency‐particle response‐mechanical motion” mechanism. These findings validate frequency modulation as an effective control strategy for high‐speed actuation in magnetic soft robots.

To evaluate the structural and functional stability after repeated reprogramming, the magnetic properties, mechanical modulus, and microstructural interface of the composite were characterized before and after 100 heating‐reprogramming cycles. VSM measurements (Figure 4j) show that the magnetic remanence increases from 5.1 to 7.9 emu/g after 100 cycles compared with the original sample, accompanied by improved saturation magnetization and coercivity. This improvement arises from internal stress relaxation and more uniform rearrangement of magnetic domains during repeated melting‐solidification cycles, which enhances interparticle magnetic coupling and leads to enhanced magnetic ordering rather than performance degradation. Uniaxial tensile test results (Figure 4k) demonstrate that the mechanical modulus remains stable with no significant reduction, confirming reliable mechanical integrity. High‐resolution SEM images (Figure S8b) show no obvious PEG leakage, phase separation, or SmFeN particle agglomeration. The interface between PEG microspheres and the PDMS matrix remains clear and well‐bonded after extensive cycling. These results confirm the excellent structural stability and durable magnetic responsiveness of the composite under repeated reprogramming.

### 2.4 Locomotion Performance Analysis and Gait Optimization

Comparative analysis of displacement‐time trajectories (Figure S10 and Movie S6) reveals clear distinctions in locomotion dynamics. The rolling gaits (plain, Rolling1; multipod‐enhanced, Rolling2) consistently achieved higher velocities than their creeping counterparts (Creeping1 and Creeping2, respectively), confirming the greater efficiency of continuous rolling compared to stepwise rhythmic body deformation. This performance was further improved by conical multipods (Figure S9), which enhanced both gait patterns: rolling kinetics were accelerated (multipod‐enhanced Rolling2 vs. plain Rolling1), and creeping displacement was increased (multipod‐enhanced Creeping2 vs. plain Creeping1).

The multipods improve locomotion through enhanced interfacial adhesion and mechanical coupling. This effect enables smoother gait transitions during rolling and more efficient force transmission in creeping, while reducing energy consumption [46, 47, 48, 49, 50, 51]. The robot can achieve a maximum speed of 1.8 BL/s (27 mm/s) in the multi‐legged rolling mode. The outstanding performance of Rolling2 demonstrates a coordinated interplay between motion mode, structural design, and kinetic performance. Specifically, during rolling motions, the conical geometry increases frictional torque through normal adhesive forces at contact points, while its low‐resistance characteristic during detachment lowers the energy barrier for flipping, thereby improving rolling efficiency. These results provide a quantitative framework for optimizing locomotion in soft‐bodied robotic systems.

To further contextualize the locomotion performance of our robot, we compared it with other recent reprogrammable magnetic soft robots. Table S1 summarizes key metrics including reprogramming material/mechanism, reprogramming temperature, response time, maximum speed (in body lengths per second, BL/s), and sensing capability (Yes/No). As shown in Table S1, our robot offers the lowest reprogramming temperature (∼39.5°C) among all the compared systems that rely on thermal actuation. This physiologically compatible temperature window (38°C–42°C) is critical for biomedical applications to avoid thermal injury to surrounding tissues. Moreover, our robot achieves a competitive maximum speed of ∼1.8 BL/s while also integrating multimodal sensing capability (capacitive sensor for environmental and self‑motion monitoring). In contrast, most existing reprogrammable magnetic soft robots either lack sensing functions or operate at much higher temperatures. This comparison clearly demonstrates the unique advantages and impact of our work.

**FIGURE 5:**
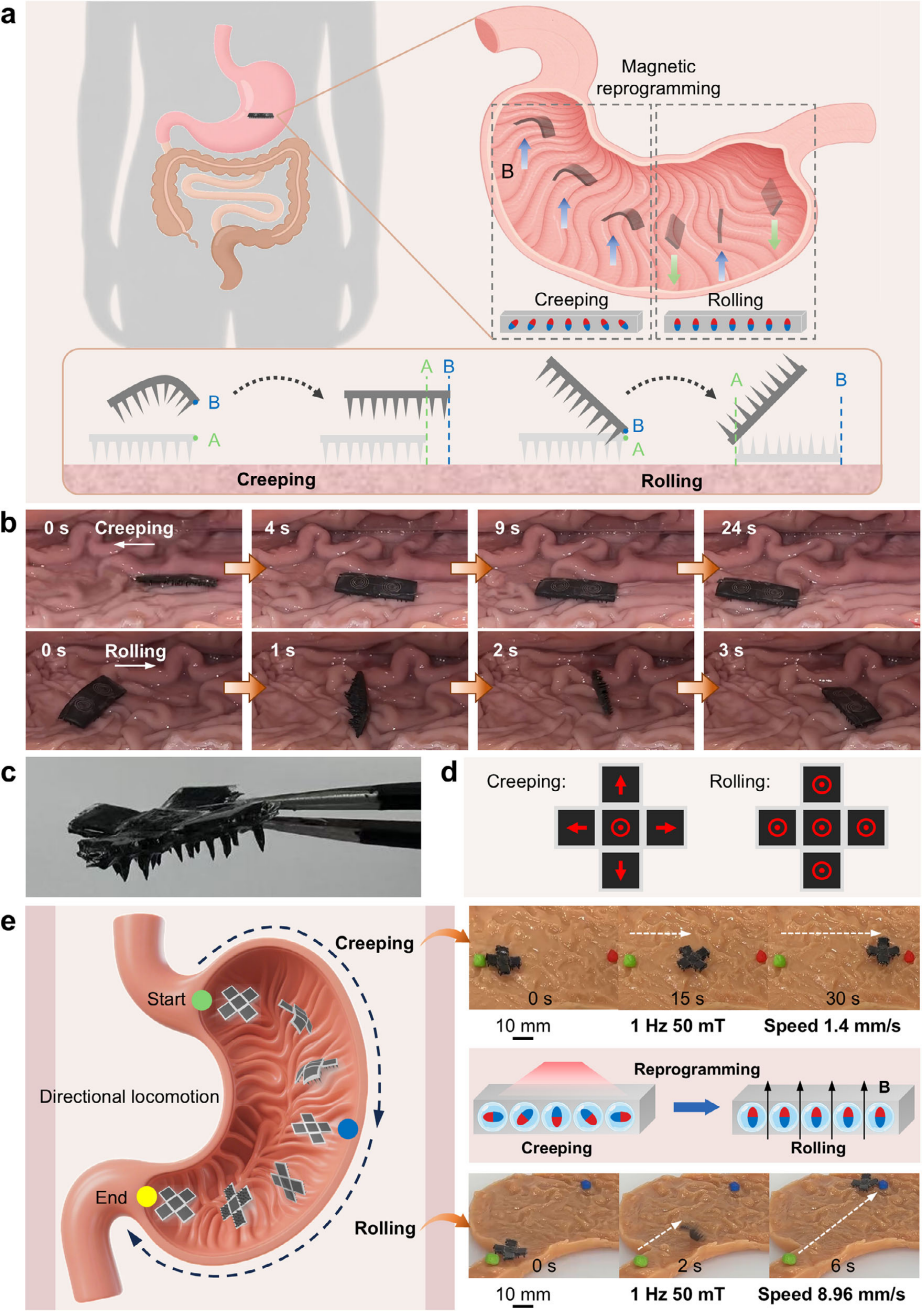
Application of the reprogrammable magnetically driven soft robot. (a) The robot's locomotion in the gastric environment (A: starting point, B: end point). (b) Creeping/rolling states of the robot. (c) Detailed diagram of the robot's conical feet. (d) Distribution of internal magnetic domains in the robot's creeping/rolling states. (e) Creeping and rolling motion of the robot on the stomach model.

Locomotion characterization of the conical multi‐legged magnetically driven soft robot within a silicone gastric model demonstrates a synergistic structure‐actuation‐environment interaction mechanism. Figure 5 presents a targeted intervention scenario in the digestive tract, where coordinated NIR heating and magnetic fields selectively activate either creeping and rolling locomotion modes (Figure 5b). The mechanical principles underlying these distinct gaits are delineated in Figure 5a rolling propulsion is achieved through periodic body flipping facilitated by alternating adhesion of the multi‐legged structure to the gastric wall, while creeping involves rhythmic body bending and recovery (Figure S11) with the multi‐legged architecture maintaining directional stability. Similarly, we fabricated a cross‐shaped soft robot with a conical multi‐legged structure via magnetic module assembly (Figure 5c), which can also achieve distinct locomotion modes through magnetic reprogramming (Figure 5e and Movie S7). Figure 5d elucidates the corresponding magnetic control strategy, where creeping is driven by alternating axial/radial magnetic domains enabling cyclic reorientation, while rolling is sustained through radially aligned domains producing continuous rotation. This programmable magnetic domain configuration enables precise locomotion mode switching. The multi‐legged structure enhances interfacial coupling via adhesion and directional friction, synergizing with magnetic domain‐mediated deformation to achieve controlled navigation across the complex gastric topography. While creeping generates displacement through magnetically‐induced postural adjustments, rolling optimizes motion efficiency through maintained magnetic alignment. This integrated magnetics‐structure‐function paradigm advances targeted digestive tract robotics by combining bioinspired adhesion with reprogrammable magnetic actuation, establishing a multidimensional experimental foundation for clinical translation of magnetically driven soft robots.

### 2.5 Fabrication of Integrated Actuation‐Sensing System

**FIGURE 6:**
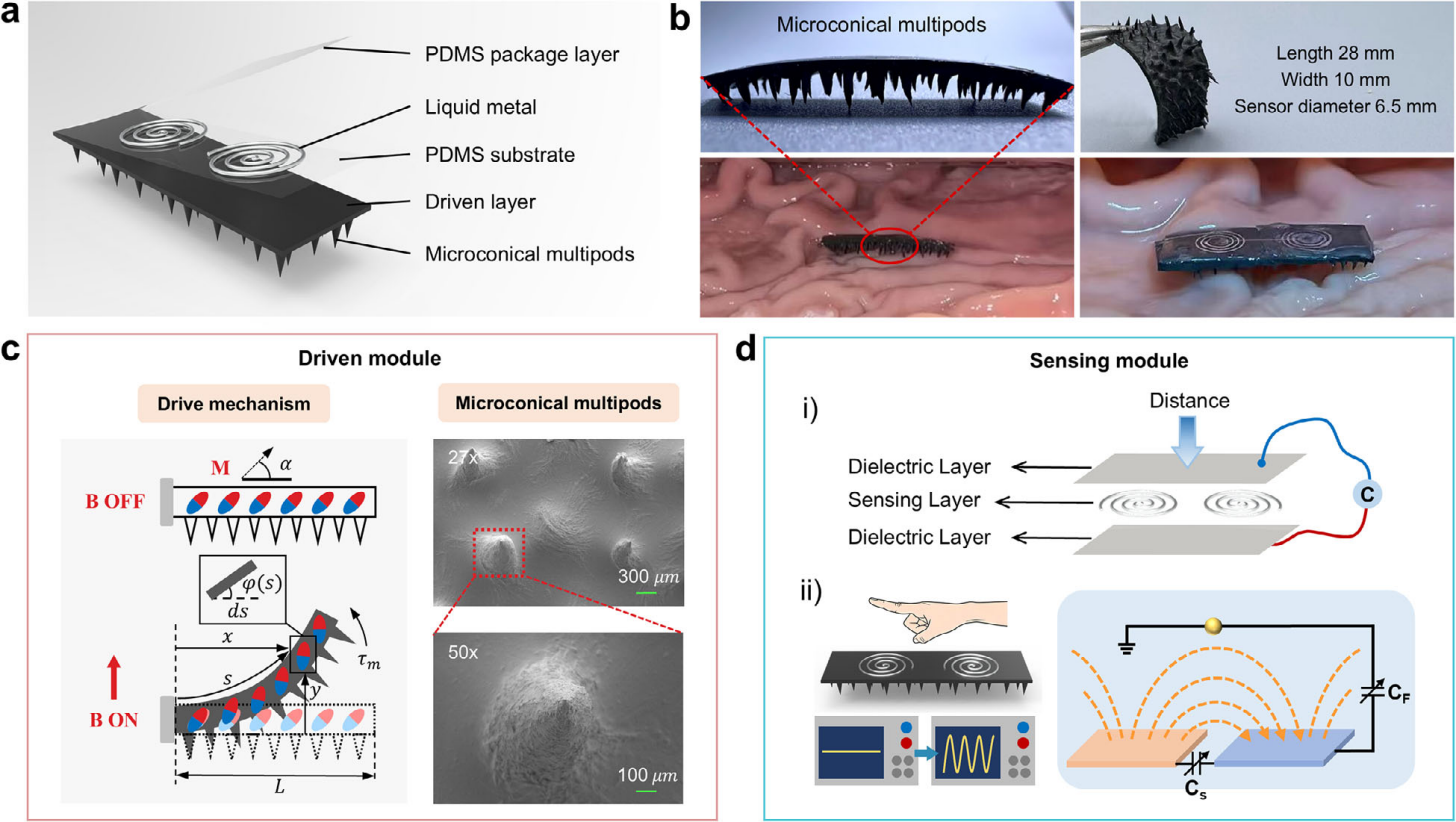
(a) The robot's driven and sensing modules. (b) Overall dimensions and structural morphology of the robot. (c) Principle of the robot's driving structure and microscopic SEM image. (d) Structure (i) and principle (ii) of the robot's sensing part.

Microelectronic printing enables direct integration of a liquid metal Archimedean spiral capacitive sensor [52, 53, 54] with the micro‐conical multipod soft robot, achieving unified actuation‐sensing functionality (Figure 6a). The sensing module, comprising the spiral conductor embedded in a PDMS encapsulation, simultaneously monitors external environmental changes and self‐motion states through capacitance variations governed by the equation $C\;=\frac{\varepsilon \cdot A}{d}$ [55, 56], where changes in dielectric constant (ε) and electrode spacing (*d*) transduce multiphysical signals into measurable electrical responses (Figure 6d and Figure S12). Concurrently, the actuation module achieves directional locomotion under magnetic guidance, assisted by interfacial coupling from the micro‐conical multipods (Figure 6c).

However, it must be noted that in the current prototype, the capacitive sensor is connected via wires for data acquisition. This physical tether inevitably introduces additional drag and stiffness, which compromises the untethered advantage of magnetic soft robots. To quantitatively assess this effect, we performed control experiments comparing the locomotion speed of the robot with and without the sensor wires (Movie S8). As shown in Figure S13, the rolling speed of the robot with the sensor wires decreased by approximately 15% (from 13.60 to 11.58 mm/s) compared to the robot without the sensor wires, and the creeping speed decreased by 36% (from 1.25 to 0.80 mm/s), confirming that the tether does measurably affect mobility.

### 2.6 Comprehensive Characterization of Sensing Performance

**FIGURE 7:**
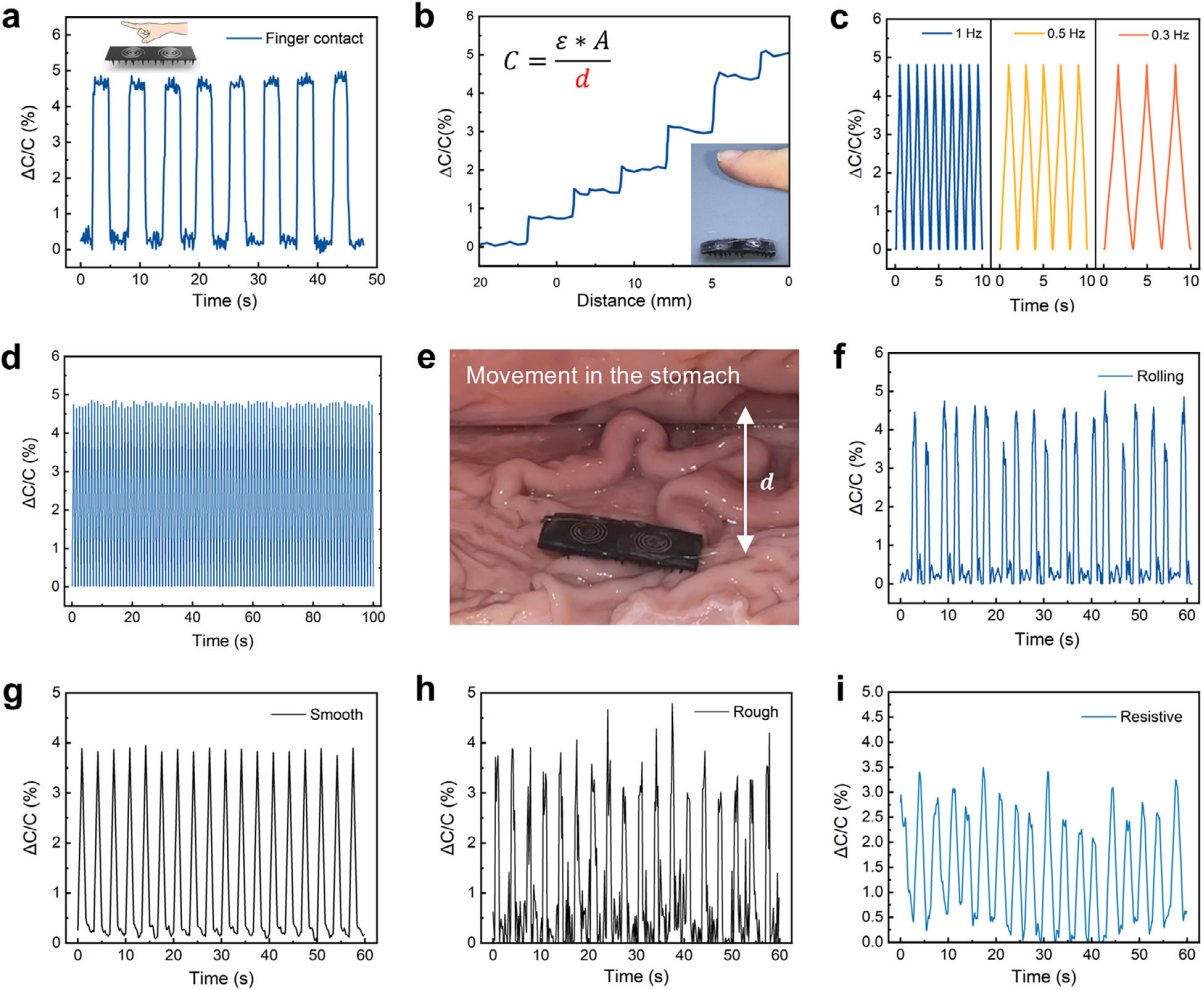
Capacitive sensing performance under varying conditions. (a) Contact/non‐contact experiments of the capacitive sensor. (b) Effect of contact distances on capacitance sensitivity. (c) Effect of contact frequencies on capacitance sensitivity. (d) Repeated contact experiments. (e) Schematic of a multi‐legged sensing robot inside a pig stomach (*d* represents the distance between the stomach wall). (f) Capacitance data of the robot in the rolling state. (g) Capacitance data of the robot creeping on smooth terrain. (h) Capacitance data of the robot creeping on rugged terrain. (i) Capacitance data of the robot creeping on smooth and resistive terrain.

The capacitive response during alternating contact and non‐contact states (Figure 7a) revealed periodic transitions between high and baseline capacitance levels, validating reliable binary state detection. Quantitative analysis of contact distance dependence (Figure 7b) demonstrated precise agreement with the parallel‐plate capacitance model ($C\;=\frac{\varepsilon \cdot A}{d}$), establishing a fundamental relationship between proximity and electric field coupling efficiency. Frequency response characterization (Figure 7c) confirmed consistent signal integrity across 0.3–1 Hz excitation frequencies, ensuring robust operation under dynamic mechanical interactions. Cyclic testing over 100 contact cycles (Figure 7d) verified exceptional signal stability (< 2% deviation), highlighting the liquid metal microstructure's durability for extended operation. Material‐dependent sensing performance (Figure S15e) showed systematic capacitance variations across polystyrene, rubber, glass, and iron substrates, enabling discrimination based on dielectric properties. Motion‐specific signatures were clearly identified: rolling locomotion (Figure 7f and Movie S9) produced irregular yet repeatable fluctuations corresponding to intermittent foot‐surface contact; smooth terrain creeping (Figure 7g) exhibited sharp periodic peaks; rugged terrain (Figure 7h) generated complex aperiodic signals mimicking biological tissue topography; while resistive conditions (Figure 7i) resulted in attenuated waveforms quantifying external load effects (Figure S14). Different terrains and motion modes (Movie S10) induce distinct capacitance variations, collectively demonstrating the sensor's capability to capture nuanced mechanical interactions in diverse operational scenarios.

The integrated capacitive sensing system successfully demonstrates multifunctional perception capabilities essential for autonomous soft robots. The sensor provides simultaneous monitoring of environmental properties, such as material composition, surface topography, and mechanical constraints, while continuously tracking self‐motion states through distinct locomotion signatures. This dual sensing modality, combined with stable performance across varying operational frequencies and extended testing cycles, establishes a robust foundation for closed‐loop control in biologically relevant environments. The consistent correlation between theoretical models and experimental results further validates the system's reliability for quantitative applications in minimally invasive medicine and adaptive robotics.

However, it should be clarified that in the current implementation, the sensor data are recorded and analyzed offline to identify different terrains, contact materials, and locomotion gaits. These data are not fed back in real time to automatically adjust the actuation magnetic field (e.g., changing field strength, frequency, or direction based on terrain detection). Therefore, the current level of automation is limited to open‑loop perception, rather than closed‑loop control. We agree that closing the control loop is a crucial step toward truly intelligent soft robots. In future work, we plan to integrate the sensor with a real‑time control system (e.g., using a microcontroller with a feedback algorithm) so that the robot can autonomously adapt its locomotion based on environmental sensing.

To overcome the limitation of the wired connection, we leverage the inherent advantages of the Archimedean spiral capacitive sensor from the original design. The spiral structure itself exhibits both inductive and capacitive characteristics, enabling a self‐integrated passive inductor‑capacitor (LC) resonant circuit. As a result, passive and wireless operation is achieved without the need for built‐in batteries or external inductive components. Based on this, we have developed a wireless, battery‑free sensing system, converting environmental parameters into a shift in resonant frequency, which can be wirelessly interrogated [57] and powered by an external reader. The system provides a solid foundation for remote signal transmission in complex biological lumens. As shown in Figure S15f, the wireless LC sensor can reliably discriminate different dielectric media (e.g., Fe, glass, polystyrene) through distinct resonant frequency shifts. This validation confirms the feasibility of the wireless passive sensing approach. Nevertheless, this wireless sensor has not yet been fully integrated with the soft robot for real‑time monitoring of locomotion and environmental interactions. Developing a fully wireless, integrated actuation‑sensing soft robot is a key focus of our ongoing and future work.

## 3 Conclusion

In summary, this study establishes a novel paradigm for creating multimodal magnetically driven soft robots through photothermal reprogramming technology. By leveraging the unique photothermal properties of SmFeN particles within custom‐designed PEG/SmFeN microspheres, we have developed a material system that enables selective and reversible magnetic domain reconfiguration. This core capability decouples the robot's structure from its function, allowing a single robotic entity to be reprogrammed post‐fabrication for diverse locomotion gaits and sophisticated shape transformations, as demonstrated by the superior rolling efficiency and targeted creeping in a simulated gastric environment. The integration of a liquid metal Archimedean spiral capacitive sensor via microelectronic printing creates a unified actuation‐sensing system. This system successfully demonstrates simultaneous monitoring of environmental properties, while continuously tracking self‐motion states through identifiable locomotion signatures. The stable performance over extended cycles across various operational scenarios validates its robustness for closed‐loop control in biologically relevant environments. In addition, by optimizing the PEG composition (PEG1000:PEG1500 = 1:2), we have lowered the reprogramming temperature to ∼39.5°C, which is compatible with in vivo use and avoids thermal injury to surrounding tissues. Collectively, our work provides a comprehensive materials and integration platform that synergizes spatially programmable actuation with multimodal sensing. This magnetics‐structure‐function paradigm not only addresses the longstanding limitation of fixed magnetization profiles in magnetic soft robotics but also opens new avenues for developing intelligent, adaptive systems for minimally invasive medicine and targeted therapeutic delivery. Looking forward, we will focus on implementing closed‑loop control by feeding the capacitive sensor data back to the magnetic actuation system, enabling the robot to autonomously adjust its locomotion in response to environmental changes. Another key direction is enhancing material biodegradability, integrating the wireless LC sensor into the soft robot body to eliminate the tether, and incorporating real‐time imaging functionalities to accelerate clinical translation.

## 4 Experimental Section

### 4.1 Materials

Poly(ethylene glycol) (PEG, MW = 2000 and 20 000) was purchased from Macklin. Samarium‐iron‐nitrogen (SmFeN) magnetic powder (particle size 3–5 µm) was obtained from Haopeng Magnetics Co., Ltd. Polydimethylsiloxane (PDMS, Sylgard 184) was supplied by Dow Corning. Gallium‐indium‐tin (GaInSn) liquid metal alloy (melting point 6°C) was acquired from Dingguan Metal Technology Co., Ltd. All chemicals were used as received without further purification.

### 4.2 Fabrication of Magnetic Microspheres and Soft Robots

Magnetic microspheres were prepared by mixing polyethylene glycol (PEG) and samarium‑iron‑nitrogen (SmFeN) at a 1:1 mass ratio, then compounded with polydimethylsiloxane (PDMS) at a 3:4 ratio to form a flexible composite with uniform magnetic dispersion. Robot structures were fabricated via direct‑write 3D printing. During printing, initial magnetic programming combined with curing treatment enabled synergistic structure‑function design. A pulsed magnetic field oriented magnetic domains during curing to establish preset magnetization patterns.

### 4.3 Magnetic Programming Method

The constructed magnetization experimental platform comprises the following equipment: a clamp‐type magnetizer QS10‐7 (Shanghai Tianduan Industrial Co., Ltd.) and a programmable DC power supply (Meansi MAINS Corporation, MSP6500 series). The positive and negative terminals of the electromagnet were connected to the corresponding terminals of the DC power supply. The fabricated actuator was positioned between the two electromagnets in the working area according to the required magnetization direction. The power was then turned on, with the current set to 2.5 A and voltage to 38 V. Overvoltage/overcurrent/overload protections were configured at 110 V, 6 A, and 100 W, respectively. The magnetization process was completed after approximately 10 s of electrification.

### 4.4 4D Printing

The magnetically reprogrammable composites were fabricated using a Hyrel 3D printer (SDS‐10) equipped with a high‐torque mechanical extrusion system. The printing process featured integrated temperature control that enabled simultaneous printing, curing, and initial magnetic programming. System operation was controlled via G‐code with optimized parameters including single layer thickness, 6 mm/s printing speed, and regulated platform temperature, allowing fabrication of complex architectures with controlled magnetic domain alignment.

### 4.5 Liquid Metal Capacitive Sensor Fabrication

The Archimedean spiral capacitive sensor was fabricated using a dispensing printer (Shanghai Zhongbin Technology Co., Ltd.). The conductive ink was prepared by mixing gallium‐indium‐tin liquid metal (melting point 6°C) with sodium polyacrylate at 5.25% mass ratio. Both the substrate and encapsulation layers were fabricated using PDMS. The sensor structure was directly printed onto the PDMS substrate, followed by spin‐coating PDMS encapsulation layer and thermal curing at 80°C for 1 h.

### 4.6 Magnetic Actuation System

The magnetic actuation system employed a permanent magnet for motion control (intensity: 0–200 mT), combined with a Multi‐Axis Magnetic Field Control System (developed jointly with Shanghai Tiandian Magnetic Electric Co., Ltd.) for stable frequency‐controlled motion experiments. This software‐enabled system supported customizable magnetic field waveforms, including adjustable waveform type, frequency, phase, and peak values, allowing precise control over robot locomotion parameters.

## Author Contributions

L.X. conceived the idea and concept of the project. L.Y. performed the experimental procedures. L.X., L.Y., and B.W. wrote the article. All authors participated in the data collection, data analysis, and article proofreading.

## Conflicts of Interest

The authors declare no conflicts of interest.

## Supporting information




**Supporting File 1**: advs75817‐sup‐0001‐SuppMat.docx.

**Supporting File 2**: advs75817‐sup‐0002‐Movie S1.mp4.

**Supporting File 3**: advs75817‐sup‐0003‐Movie S2.mp4.

**Supporting File 4**: advs75817‐sup‐0004‐Movie S3.mp4.

**Supporting File 5**: advs75817‐sup‐0005‐Movie S4.mp4.

**Supporting File 6**: advs75817‐sup‐0006‐Movie S5.mp4.

**Supporting File 7**: advs75817‐sup‐0007‐Movie S6.mp4.

**Supporting File 8**: advs75817‐sup‐0008‐Movie S7.mp4.

**Supporting File 9**: advs75817‐sup‐0009‐Movie S8.mp4.

**Supporting File 10**: advs75817‐sup‐0010‐Movie S9.mp4.

**Supporting File 11**: advs75817‐sup‐0011‐Movie S10.mp4.

## Data Availability

The data that support the findings of this study are available from the corresponding author upon reasonable request.
