# Supplementary material for: A Multimodal Magnetically Driven Soft Robot With Integrated Actuation‐Sensing Based on Photothermal Reprogramming Technology
Source: Adv Sci (Weinh). 2026 May 22:e75817. Online ahead of print. doi: 10.1002/advs.75817 (PMC13336126; doi:10.1002/advs.75817)
Supplement: Supplementary file 1 — Supporting File 1: advs75817‐sup‐0001‐SuppMat.docx. [file ADVS-9999-e75817-s005.docx]

**Supporting Information**

**A Multimodal Magnetically Driven Soft Robot with Integrated Actuation-Sensing Based on Photothermal Reprogramming Technology**

*Liu Yang, Yuliao Dong, Kangning Li, Cheng Jing, Ben Wang, Lin Xu**

L. Yang, Y. Dong, K. Li, C. Jing, L. Xu

School of Mechanical Engineering, Jiangsu University, Zhenjiang, 212013, P. R. China

E-mail: 1000003037@ujs.edu.cn

B. Wang

College of Chemistry and Environmental Engineering, Shenzhen University, Shenzhen, 518061, P. R. China

**Note S1. Magneto-Elastic Bending Model**

As shown in Fig. 5c, when an external magnetic field acts on a magnetic material, the internal magnetic particles undergo reorientation, leading to bending deformation. The conical multi-legged soft robot experiences both magnetic force and torque under external magnetic fields. In a uniform magnetic field, magnetic objects are subjected to negligible net force but significant torque^[1-2]^, expressed as:

$\tau_{m} = V_{m}\boldsymbol{M} \times\boldsymbol{B}$ (1)

where $V_{m}$, $\boldsymbol{M}$, and $\boldsymbol{B}$ represent the volume of the magnetic material, its magnetization vector, and the magnetic flux density, respectively. The deformation can thus be described using a magneto-elastic bending model.

For modeling purposes, the leg frame is defined as $\boldsymbol{L} = \{x_{l} y_{l} z_{l}\}$, with $x_{l}$, $y_{l}$, and $z_{l}$ along the leg’s length, width, and thickness directions, respectively. The magnetization of the robot actuator can be expressed as:

$\boldsymbol{M} = M{[cos\alpha sin\alpha0]}^{T}$ (2)

where $\boldsymbol{M}$ and $\alpha$ denote the magnitude and angle of magnetization. The magnetic soft actuator is modeled as a cantilever beam fixed at one end^[3]^ and deformed by external magnetic torque at the free end. Assuming small deflections, the bending moment follows the Euler-Bernoulli beam equation:

$\tau_{m}A\left( s \right)=EI\frac{\partial^{2}\varphi}{\partial s^{2}}$ (3)

Here, $A$ is the cross-sectional area, $E$ is Young’s modulus, $I$ is the moment of inertia, $s$ is the distance along the leg axis, and $\varphi= dy_{l}/dx_{l}$ is the angular deflection. Assuming a magnetic field $\boldsymbol{B} ={[0 B 0]}^{T}$ parallel to the $y_{l}$-axis at a given instant, the boundary conditions are $y_{l}(0) = 0$ and $y_{l}(L) = \pi/2 - \alpha$. Combining Eqs. (1)–(3), the deflection profile is derived as:

$y_{l}= \frac{mA{L_{l}}^{3}B}{\kappa^{3}EI}sin(\frac{\kappa}{L}x)$ (4)

where $\kappa= \pi/2 - \alpha$ is the complement of $\alpha$. This confirms that deformation amplitude increases with magnetic flux density $B$.

**Note S2. Magnetic Driving Theory**

A magnetic object in a general magnetic field may experience either magnetic force, magnetic torque, or both^[4-5]^, depending on the field configuration. The magnetic force $F_{m}$ and magnetic torque $\tau_{m}$ are given by^[6-7]^:

$F_{m} = (M\cdot\nabla)B$ (5)

$\tau_{m} = M \times B$ (6)

where $M$ is the net magnetization of the object and $B$ is the magnetic flux density. The magnetization $M$ represents the volumetric density of magnetic dipole moments. For a volume element $\Delta V$ containing multiple dipoles, $M$ is defined as:

$M = \frac{\sum_{i=1}^{n} \mu_{m_{i}}}{\Delta V}$ (7)

Magnetic flux density $B$ and magnetic field strength $H$ are related through the constitutive relation^[8-9]^:

$B = \mu_{0}(H+M)$ (8)

where $\mu_{0}$ is the vacuum permeability, $H$ is the external magnetic field vector.

In SI units, $B$ is measured in tesla (T) or gauss (Gs), with $1T ={10}^{4}Gs$. This formulation allows a unified description of magnetic actuation, linking material magnetization to deformation response under programmable field control.

**Note S3. Working Principle of Clamp-type Magnetizer**

A magnetizer is a device used for magnetizing magnetic materials. Its working principle employs a clamp-type magnetic core to hold the magnet, through which magnetic flux is conducted to achieve full magnetization of the material. Specifically, the operation of the clamp-type magnetizer proceeds as follows: First, the target magnet is secured between the magnetic cores. A controlled current is then supplied to the core via a power source, generating a strong magnetic field. This field is transmitted through the core to the magnet, inducing magnetization and thus accomplishing the magnetization process. The QS-10-7 clamp-type magnetizer exhibits high magnetic strength, stability, and uniformity, making it suitable for magnetizing various types of permanent magnets, motors, magnetic assemblies, and magnetic steels. Additionally, it offers advantages such as ease of operation, straightforward maintenance, and low energy consumption. It is widely applied in fields including machinery, electronics, automotive, and medical industries.


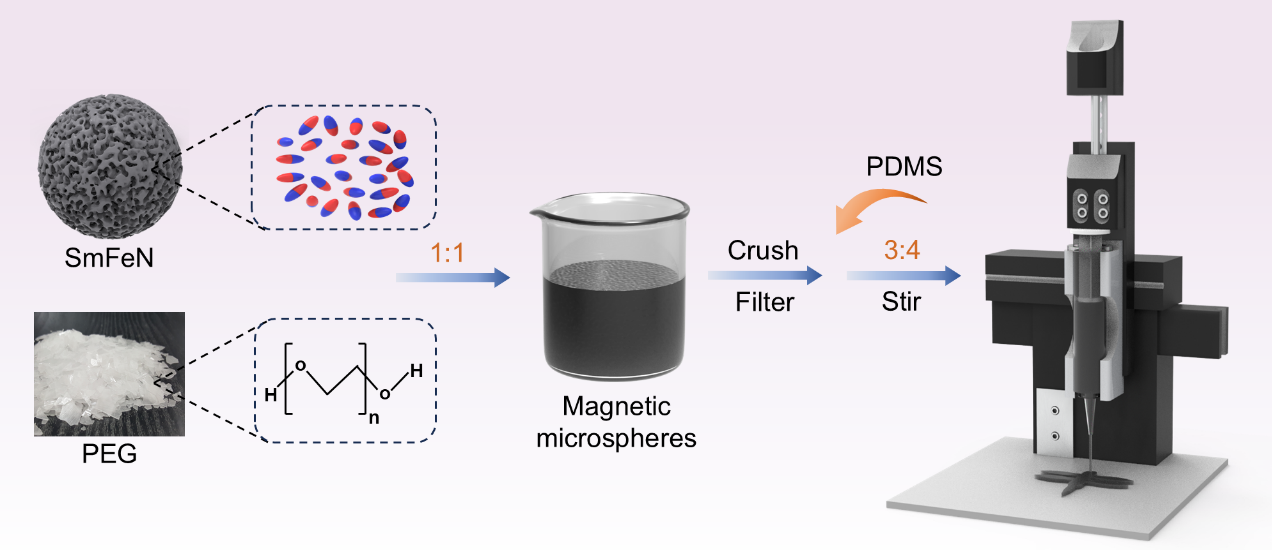


**Figure S1.** Preparation of photothermally reprogrammable materials and printing process of the robot.


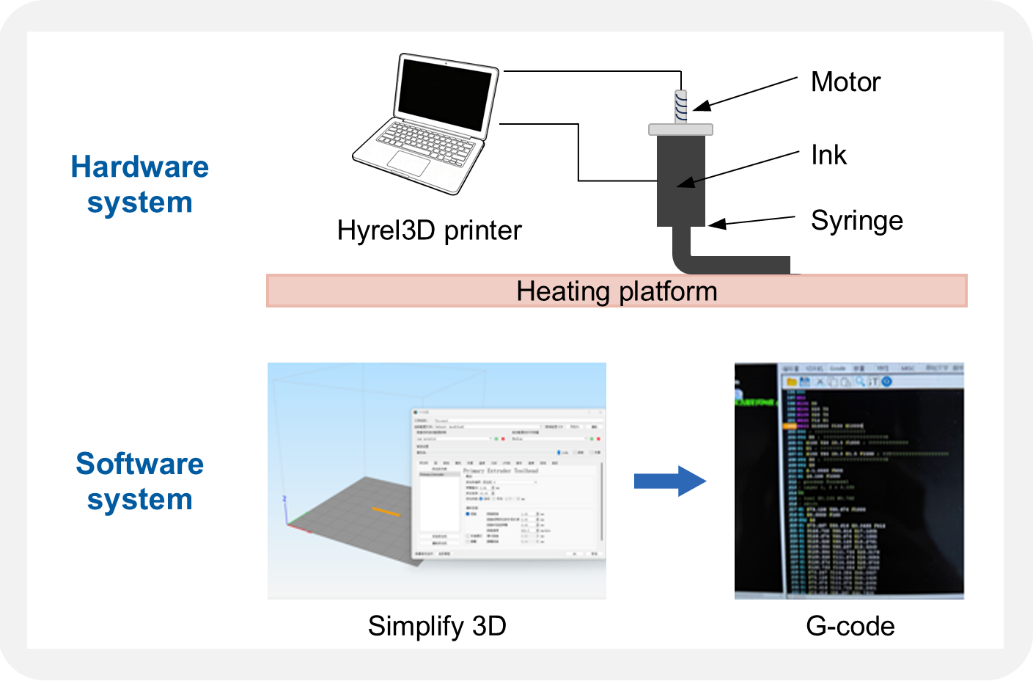


**Figure S2.** Robot printing platform.


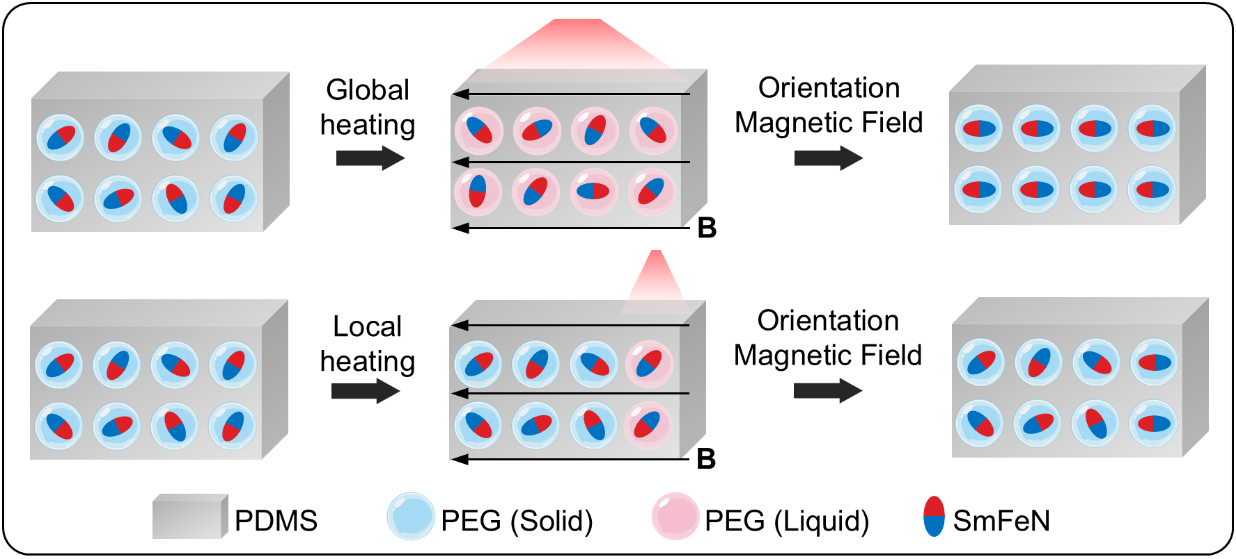


**Figure S3.** Global and local magnetic domain reconfiguration.


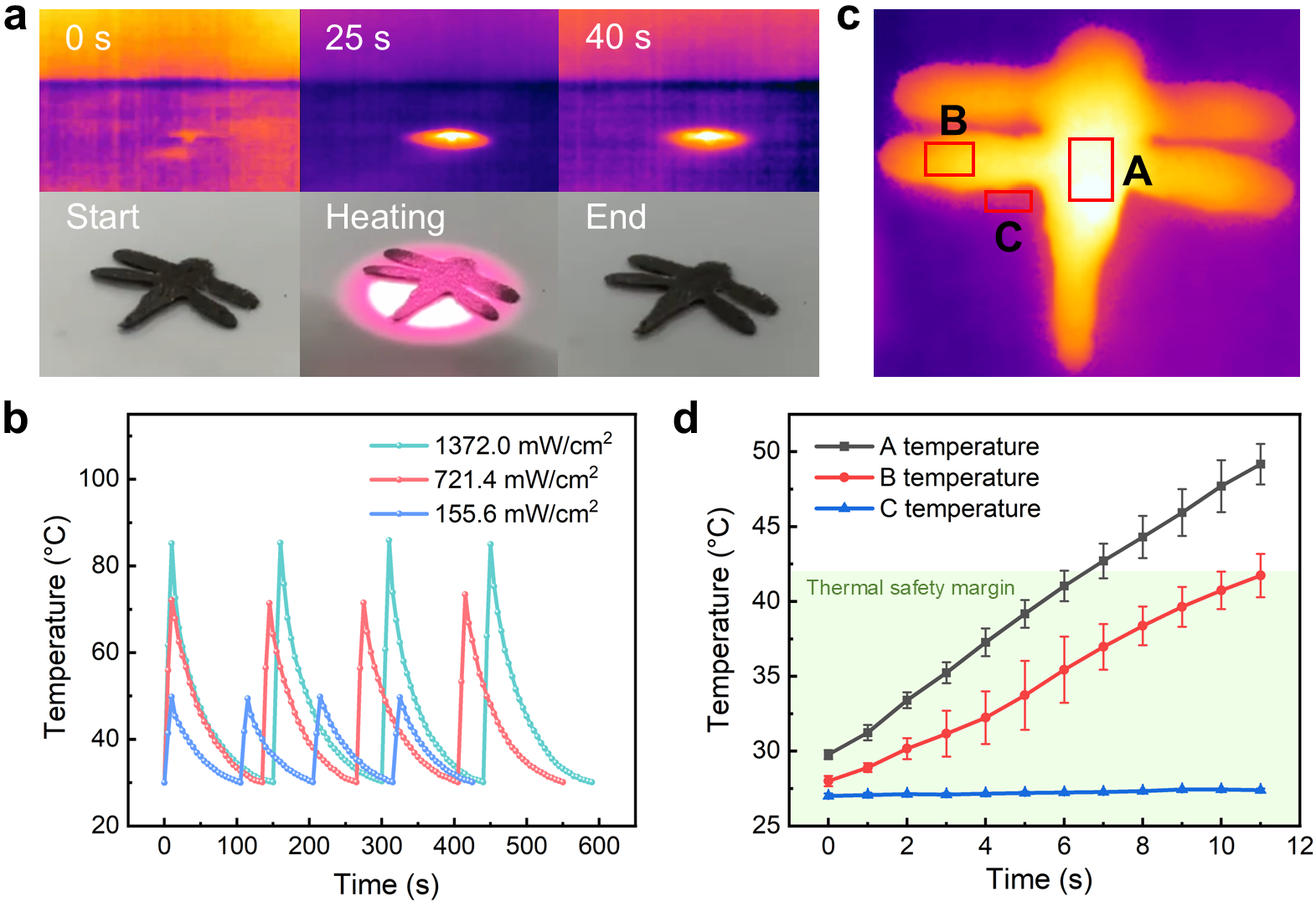


**Figure S4.** (a) The robot’s near-infrared heating and cooling under the infrared camera. (b) Heating-cooling cycle experiment of magnetic composite materials. (c) Robot thermal distribution experiment (A: core maximum temperature, B: surface temperature, C: peripheral maximum temperature). (d) Comparison of temperatures at three points A, B, C (Safe temperature threshold: 38–42°C).

**
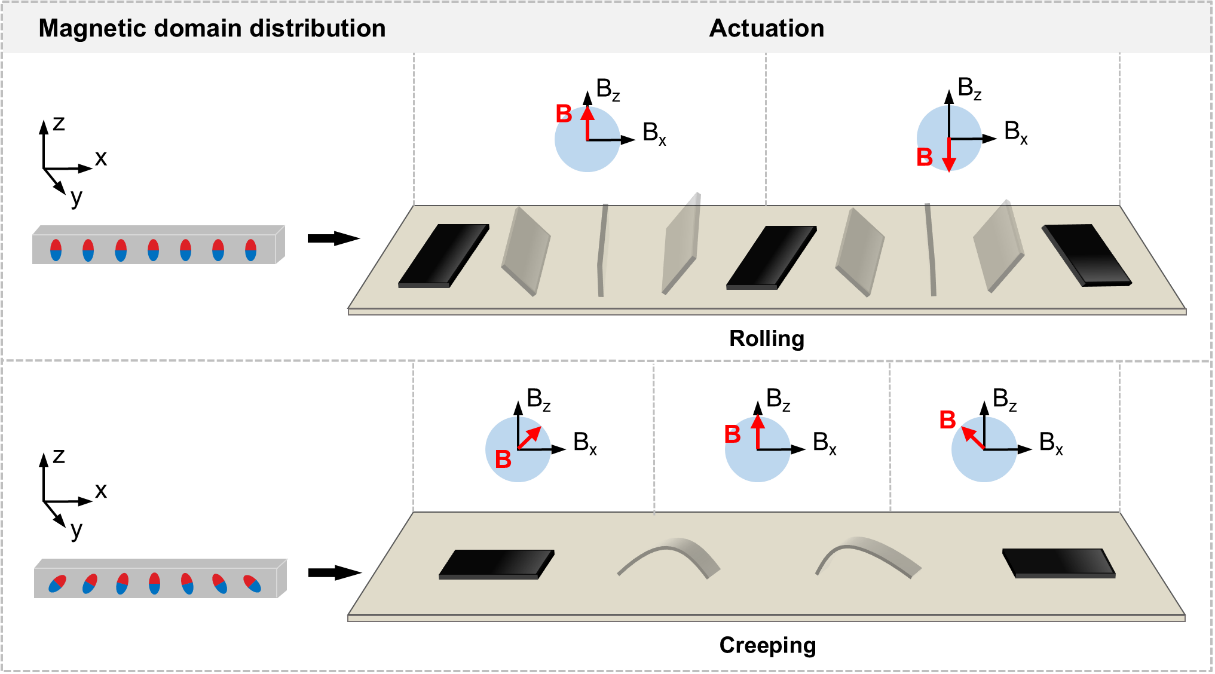
**

**Figure S5.** A strip-shaped robot demonstrating different locomotion patterns including rolling and creeping.


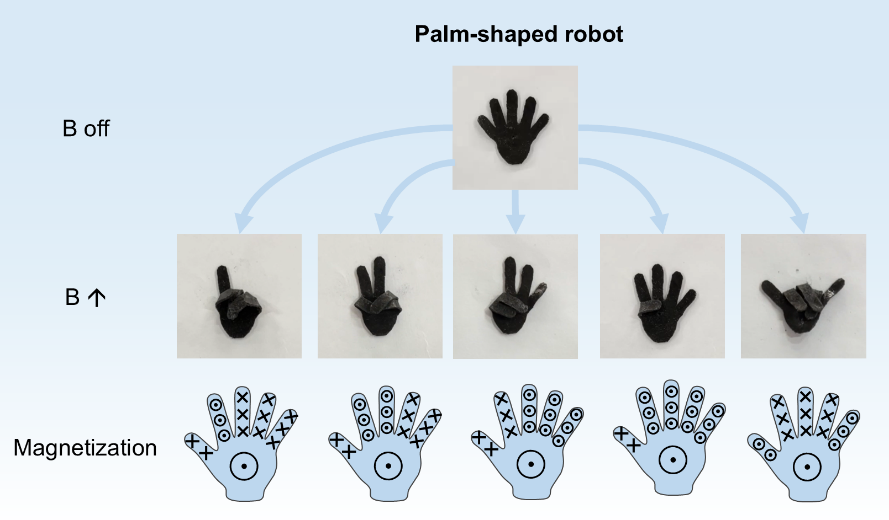


**Figure S6.** Comparison of deformation behaviors of the palm-shaped bioinspired robot before and after reprogramming.


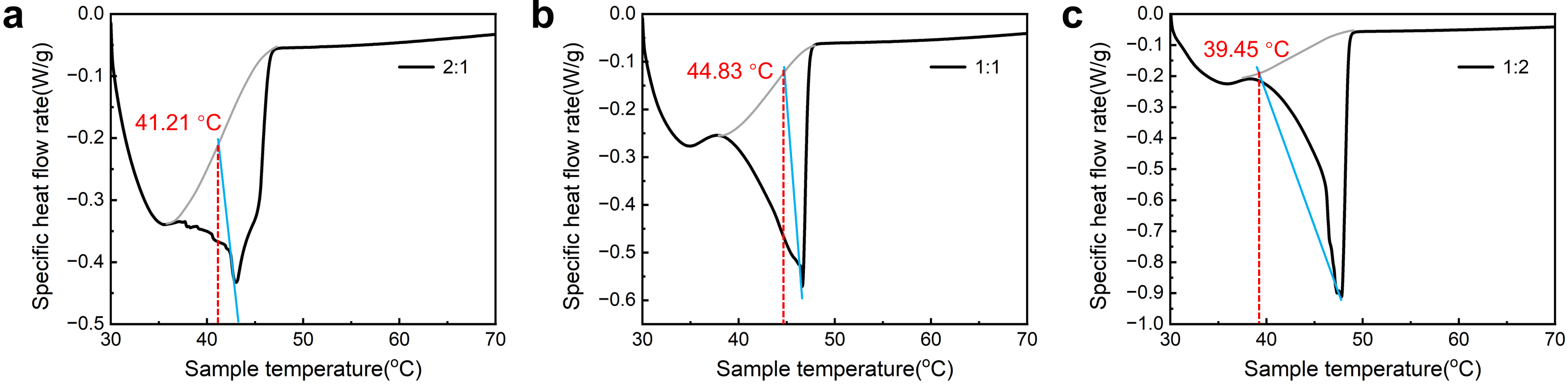


**Figure S7**. DSC results of PEG1000:1500 at different mass ratios ((a) 2:1, (b) 1:1, (c) 1:2).


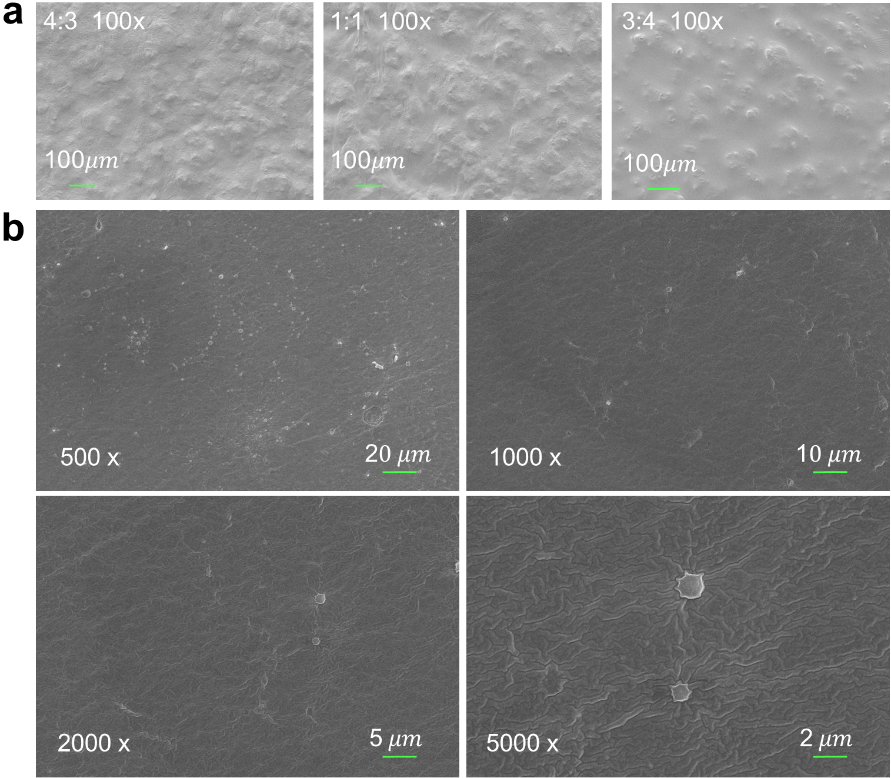


**Figure S8.** (a) Microscopic SEM images of magnetic microspheres and PDMS at three different mass ratios (4:3, 1:1, and 3:4). (b) Microstructure of the PEG/SmFeN/PDMS magnetic composite before and after 100 heating‑reprogramming cycles.


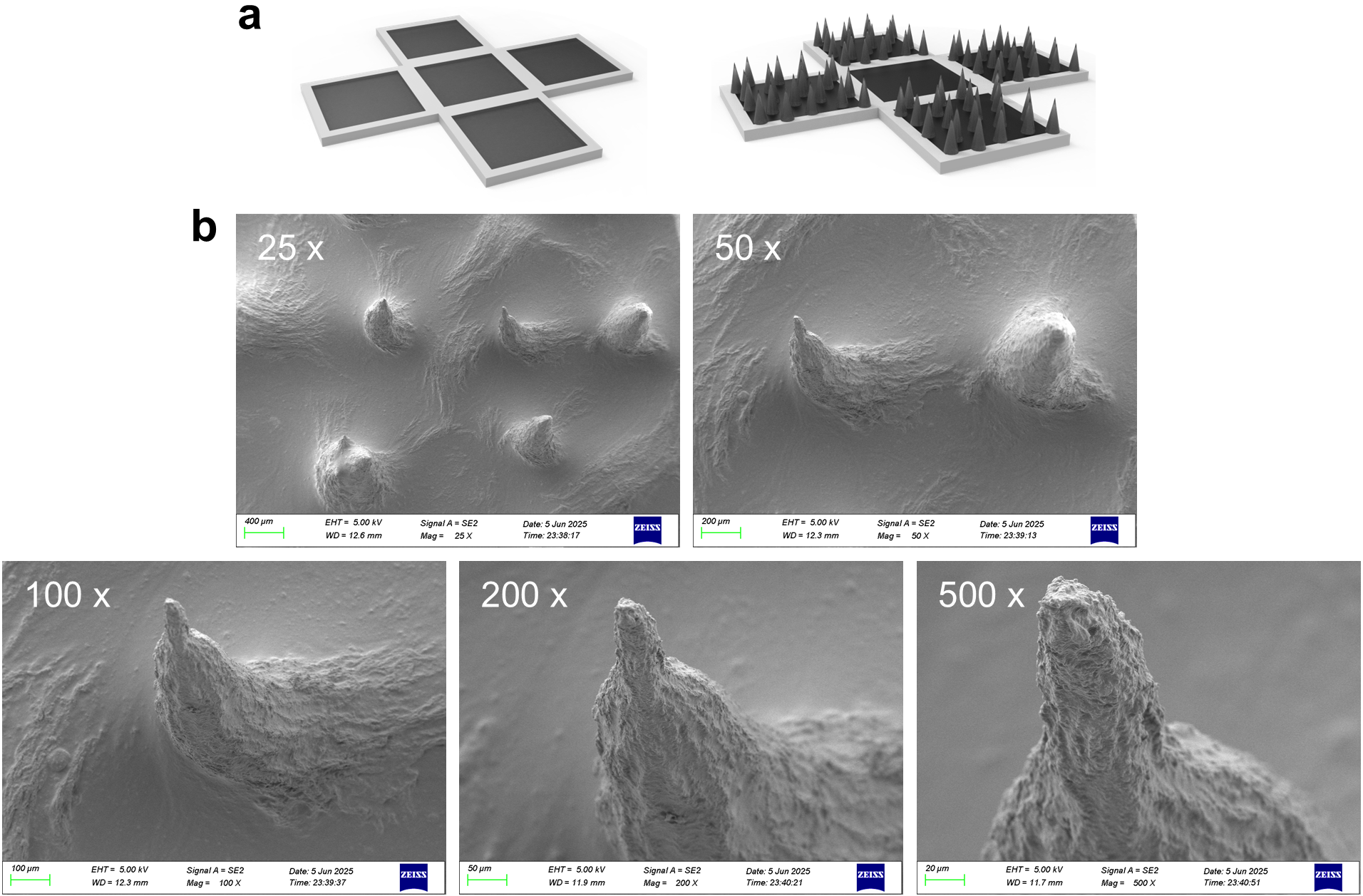


**Figure S9.** (a)Morphological comparison between plain and multipod-enhanced structures. (b) SEM micrographs: multipod structures at different ratios.


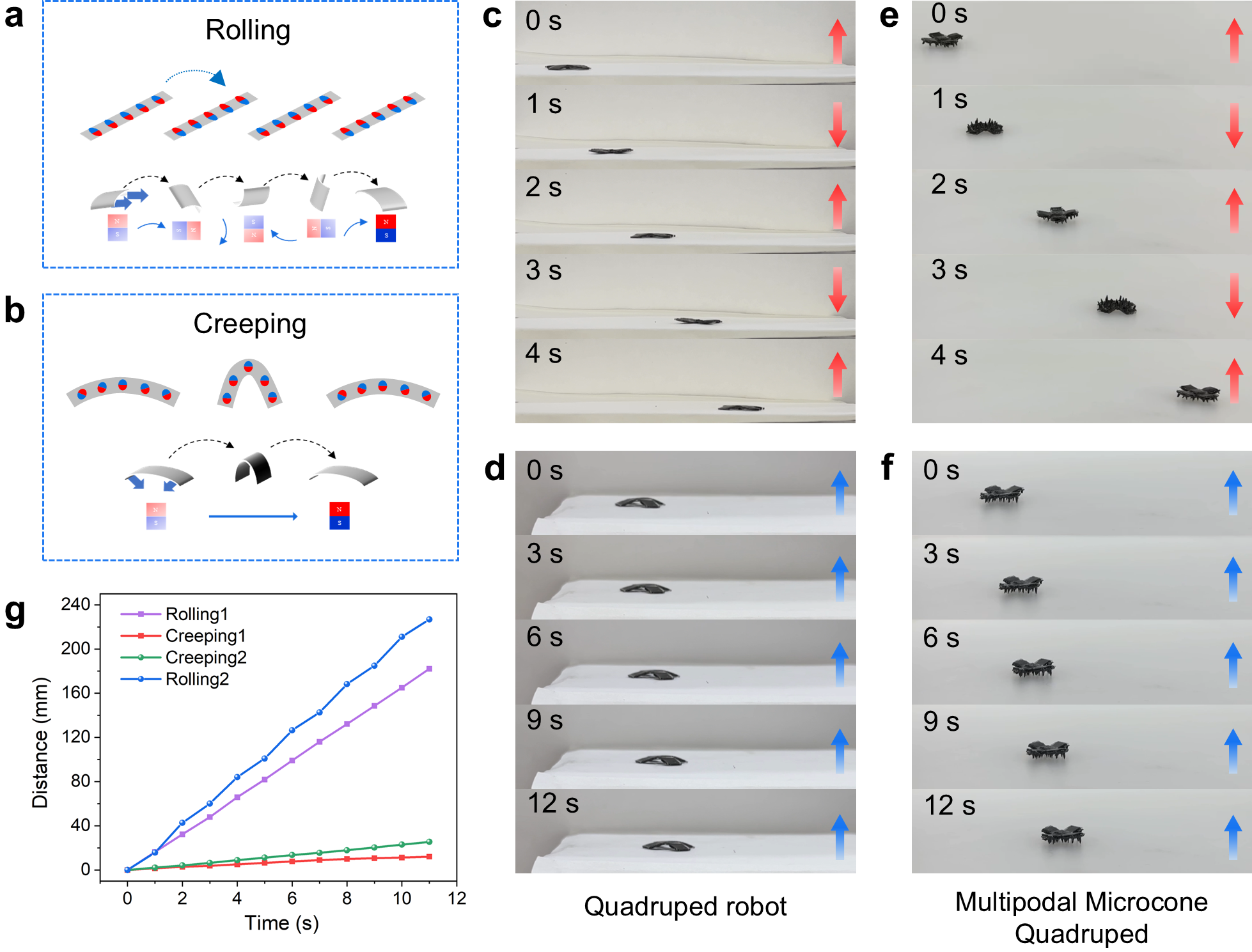


**Figure S10.** (a) Magnetic domain configuration in rolling state. (b) Magnetic domain configuration in creeping state. (c) Rolling locomotion with plain structure. (d) Creeping locomotion with plain structure. (e) Rolling locomotion with multipod-enhanced structure. (f) Creeping locomotion with multipod-enhanced structure. (g) Comparison of locomotion speeds with/without conical multi-legged structure.


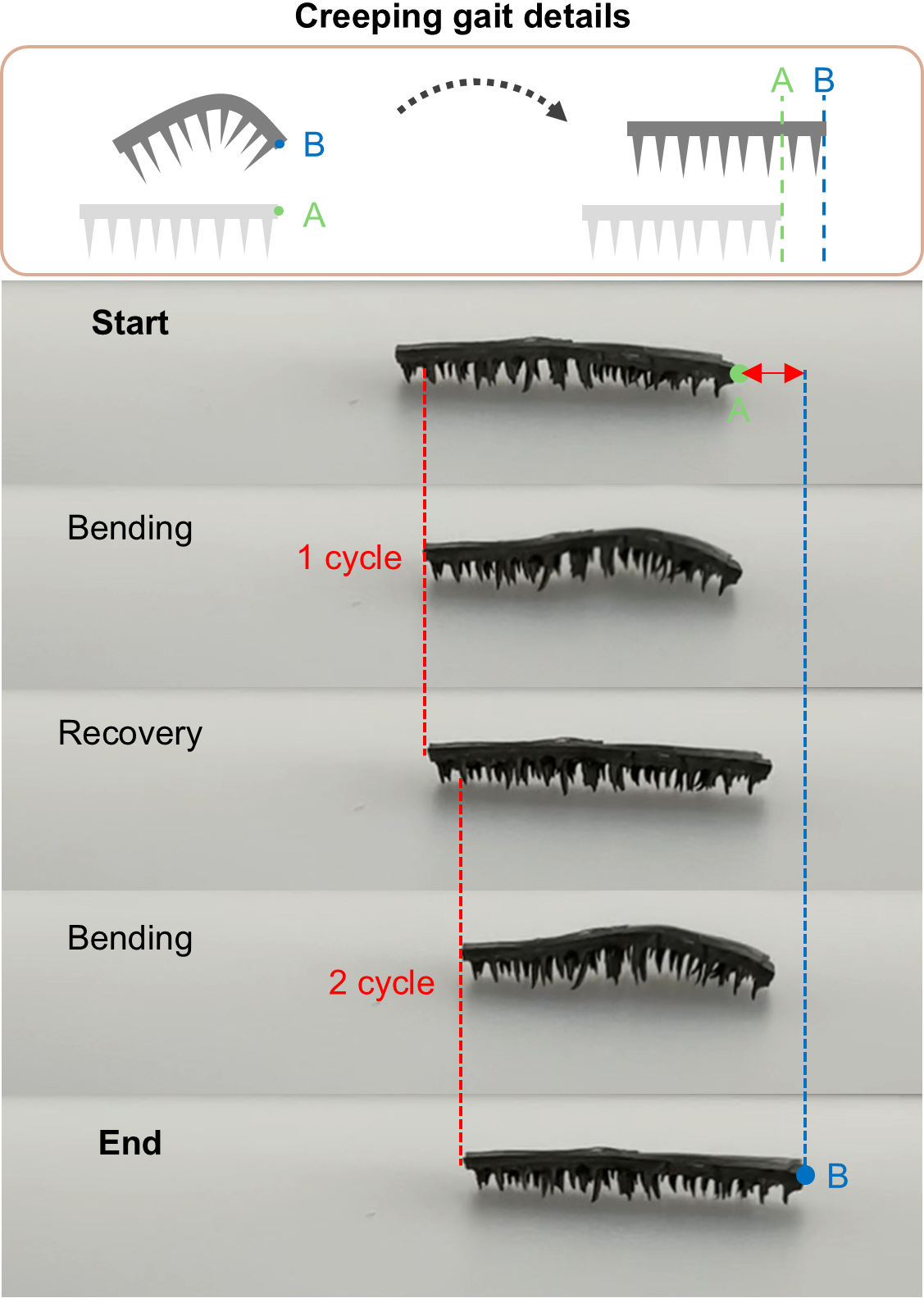


**Figure S11.** Detailed images of the robot’s ‘bending-recovery’ creeping gait within 2 cycles.


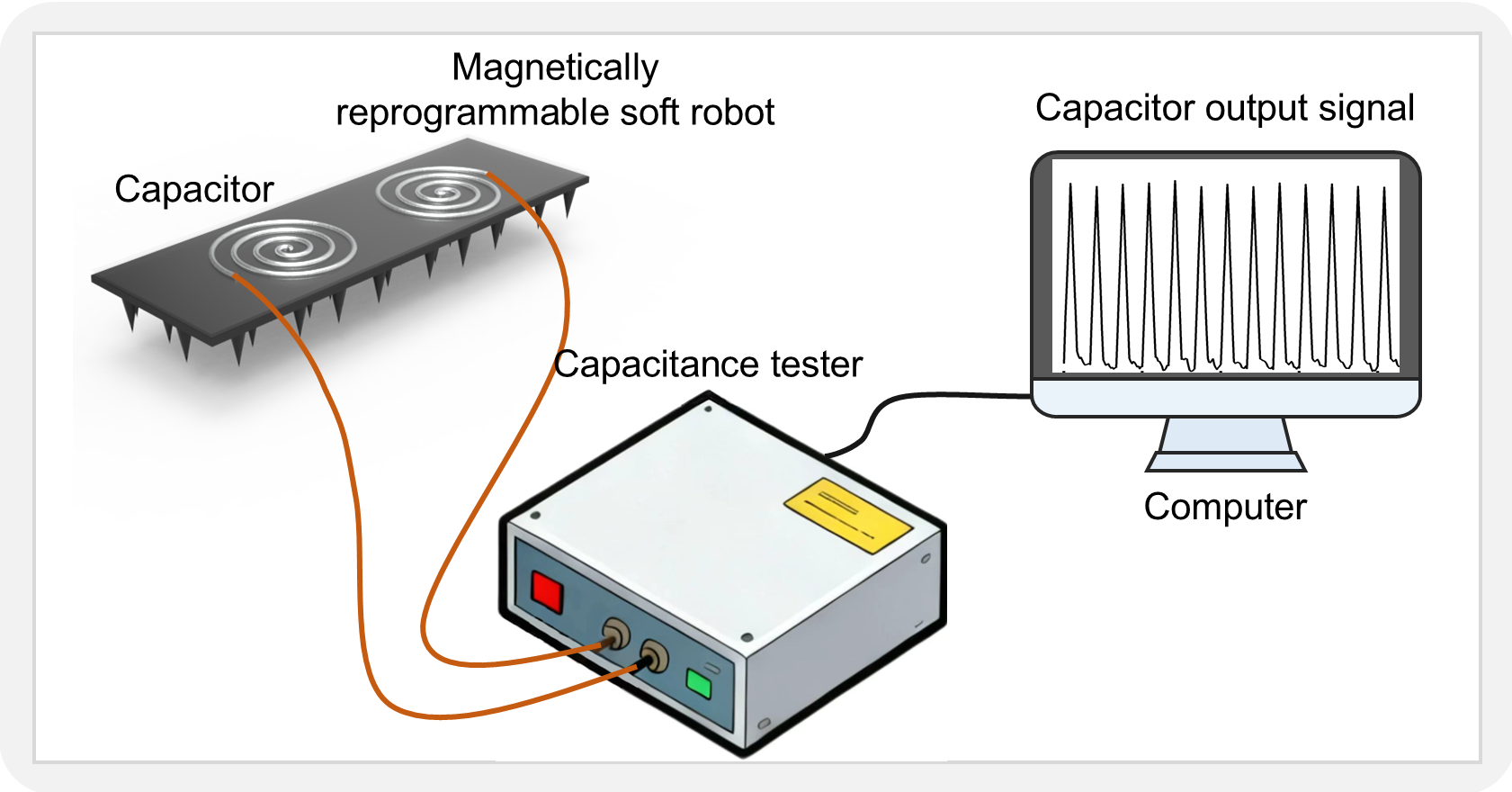


**Figure S12.** Robot sensing and transmission system.


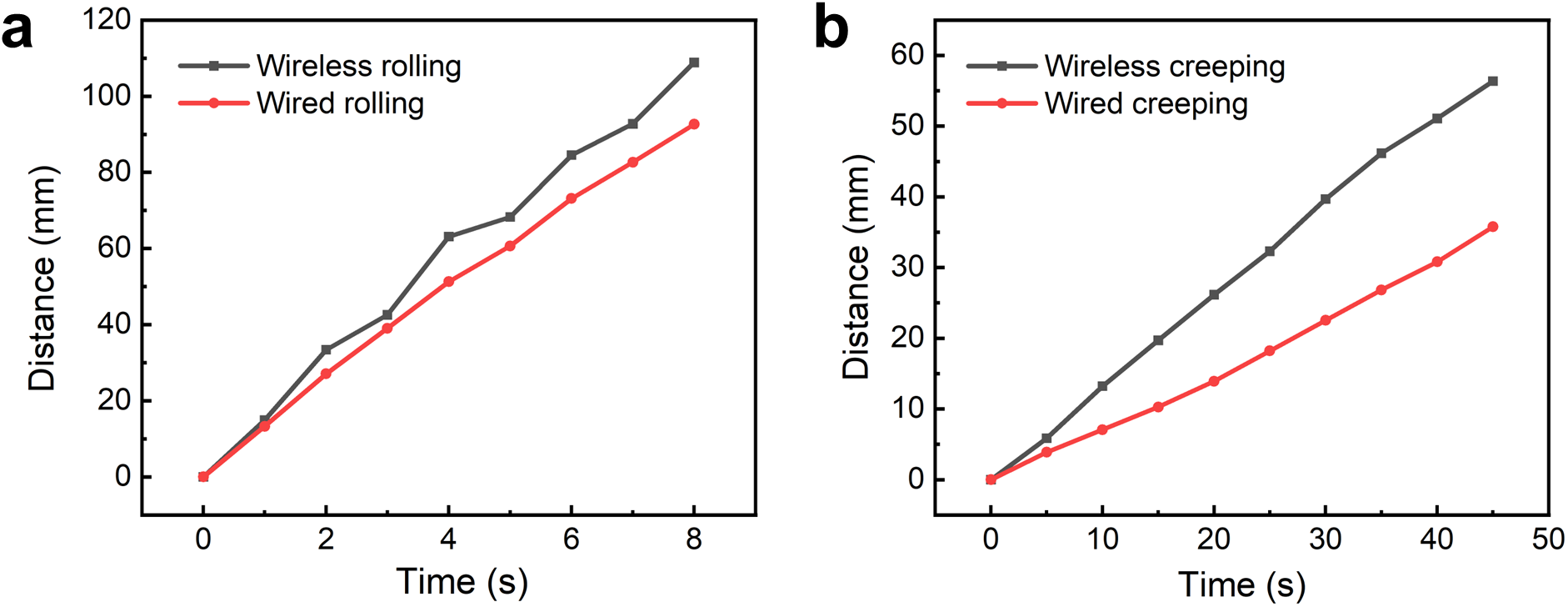


**Figure S13.** Rolling and creeping speed of the robot with and without the sensor wires.


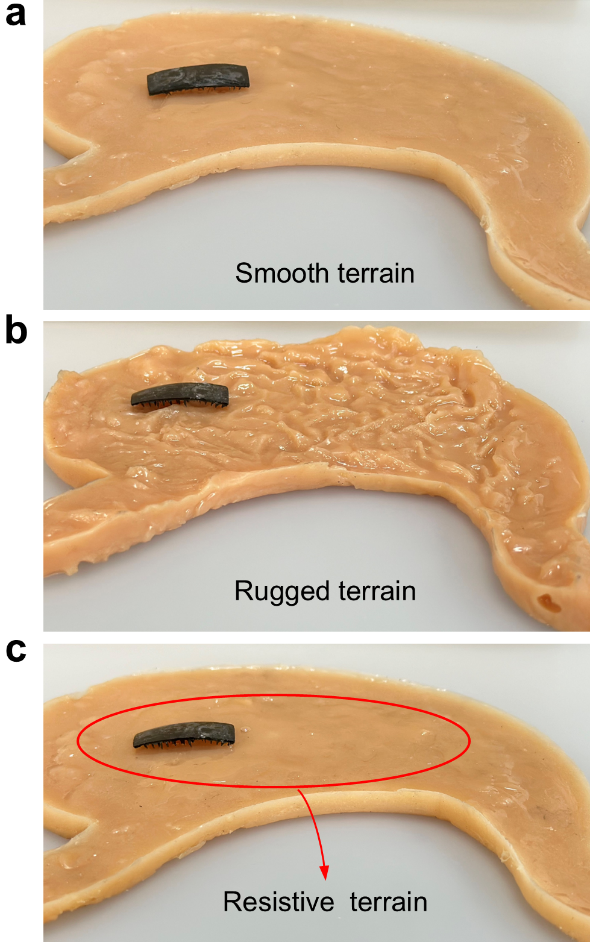


**Figure S14.** Creeping on smooth, rugged, and resistive terrains in a simulated gastric environment


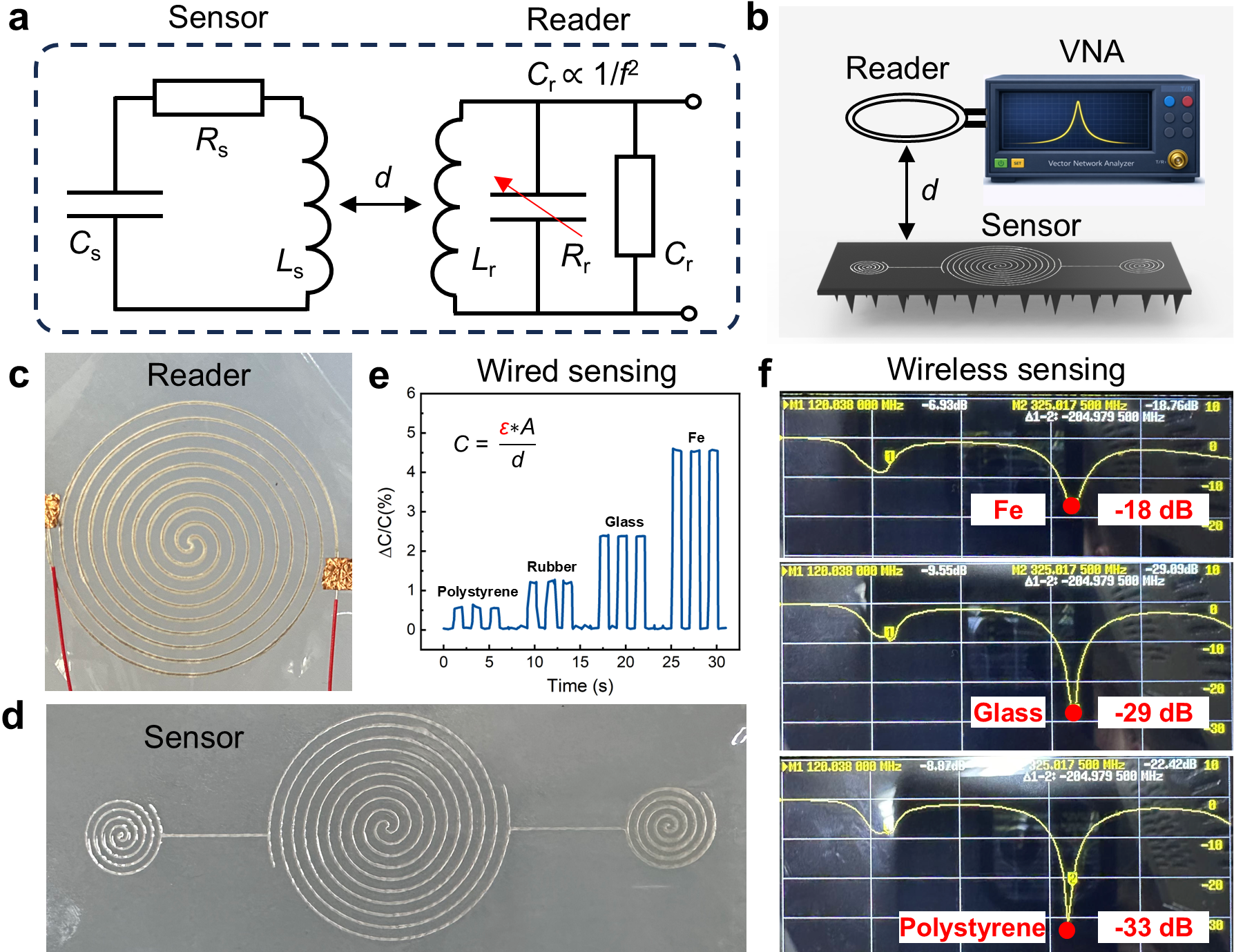


**Figure S15.** (a) Wireless sensor circuit schematic. (b) Schematic diagram of the robot’s wireless sensing. (c)(d) Structure of the wireless reader/sensor. (e)(f) Sensing results of different materials under a wired/wireless sensing structure.

**Table S1.** Comparison of reprogrammable magnetic soft robots.

| **Reference** | **Reprogramming Material/Mechanism** | **Reprogramming Temperature** | **Response Time** | **Max Speed (BL/s)** | **Sensing Capability** |
| --- | --- | --- | --- | --- | --- |
| This work | Polyethylene Glycol (PEG) | ~39.5°C | 10 s | ~1.8 BL/s | Yes |
| Sun et al., Nat. Commun., 2025^[10]^ | Ga, InPbSn, InBiSn | 30-70°C | 6 s | Not specified | Yes |
| Zhang et al., Int. J. Extrem. Manuf., 2026^[11]^ | Ethylene vinyl acetate (EVA) | ~85°C | 8-12 s | ~1.7 BL/s | No |
| Han et al., PNAS, 2025^[12]^ | Polyethylene Glycol (PEG) | ~65°C | Not specified | Not specified | No |
| Qi et al., Compos. Sci. Technol., 2022^[13]^ | Polycaprolactone (PCL) | ~70°C | Not specified | Not specified | Yes |
| Alapan et al., Sci. Adv., 2020^[14]^ | CrO_2_ | ~118°C | 5.7 s | Not specified | No |
| Han et al., Nano-Micro Lett., 2025^[15]^ | Soybean wax | ~45°C | Not specified | Not specified | Yes |
| Deng et al., Nat. Commun., 2020^[16]^ | Polycaprolactone (PCL) | ~60°C | Not specified | Not specified | No |
| Zhao et al., IEEE RA-L, 2022^[17]^ | Liquid metal | Room temperature | Without heating | Not specified | No |
| Kuang et al., Adv. Mater., 2021^[18]^ | Retro Diels-Alder (rDA) | 110-130°C | Not specified | Not specified | No |

**Supporting Videos**

**Movie S1.** Cyclic heating and cooling of the photothermal-magnetic reprogrammable material.

**Movie S2.** Shape morphing of petal structure before and after reprogramming.

**Movie S3.** Shape morphing of dragonfly robot before and after reprogramming.

**Movie S4.** Gesture transformations of palm-shaped robot before and after reprogramming.

**Movie S5.** Locomotion response under different magnetic field frequencies.

**Movie S6.** Locomotion speed with and without conical multipods.

**Movie S7.** Creeping and rolling gaits on a stomach model.

**Movie S8.** Rolling and creeping of the robot under wired and wireless conditions.

**Movie S9.** Rolling locomotion of robot with conical multipods on smooth stomach surfaces.

**Movie S10.** Rolling and creeping locomotion of a sensor-equipped robot within a pig’s stomach.

**References**

1. T. Jang, H. J. Ha, S. H. Sohn, *New Phys.: Sae Mulli* **2022**, *72*, 930.
2. J. J. Abbott, O. Ergeneman, M. P. Kummer, A. M. Hirt, B. J. Nelson, *IEEE Trans. Robot.* **2007**, *23*, 1247.
3. 003-Straight beam models: Hamilton’s principle, In *Modelling of Mechanical Systems*, Elsevier, **2005**, pp. 130–187.
4. K. Fujisaki, Ed., *004-Magnetic Material for Motor Drive Systems: Fusion Technology of Electromagnetic Fields*, Springer Singapore, Singapore, **2019**.
5. M. Brun, F. Cortés, M. J. Elejabarrieta, *J. Sound Vibration* **2025**, 119550.
6. W. Li, K.-M. Lee, *IFAC-Pap.* **2023**, *56*, 391.
7. B. J. Nelson, I. K. Kaliakatsos, J. J. Abbott, *Annu. Rev. Biomed. Eng.* **2010**, *12*, 55.
8. R. W. Brown, Y. N. Cheng, E. M. Haacke, M. R. Thompson, R. Venkatesan, *008-Magnetic Resonance Imaging: Physical Principles and Sequence Design*, 1st ed., Wiley, **2014**.
9. V. L. Safonov, *009-Nonequilibrium Magnons: Theory, Experiment, and Applications*, 1st ed., Wiley, **2012**.
10. Y. Sun, B. Sun, X. Cui, W. Li, Y. Zhang, L. He, S. Nong, Z. Zhu, J. Wu, D. Li, X. Li, S. Zhang, X. Li, M. Li, *Nat. Commun.* **2025**, *16*, 2267.
11. Q. Zhang, R. Li, Y. Tao, Y. Chen, Y. Hu, D. Wu, J. Chu, J. Li, *Int. J. Extrem. Manuf.* **2026**, *8*, 015508.
12. J. Han, S. Wang, Z. Zheng, D. Chen, W. Zhang, Z. Qu, M. Cheng, Y. Yao, M. Sitti, L. Dong, *Proc. Natl. Acad. Sci.* **2025**, *122*, e2426846122.
13. S. Qi, H. Yao, J. Fu, Y. Xie, Y. Li, R. Tian, M. Yu, H. Guo, *Compos. Sci. Technol.* **2022**, *230*, 109789.
14. Y. Alapan, A. C. Karacakol, S. N. Guzelhan, I. Isik, M. Sitti, *Sci. Adv.* **2020**, *6*, eabc6414.
15. S. Han, J.-W. Shin, J. H. Lee, B. Li, G.-J. Ko, T.-M. Jang, A. Dutta, W. B. Han, S. M. Yang, D.-J. Kim, H. Kang, J. H. Lim, C.-H. Eom, S. J. Choi, H. Cheng, S.-W. Hwang, *Nano-Micro Lett.* **2025**, *17*, 152.
16. H. Deng, K. Sattari, Y. Xie, P. Liao, Z. Yan, J. Lin, *Nat. Commun.* **2020**, *11*, 6325.
17. R. Zhao, H. Dai, H. Yao, IEEE Robot. *Autom. Lett.* **2022**, *7*, 4535.
18. X. Kuang, S. Wu, Q. Ze, L. Yue, Y. Jin, S. M. Montgomery, F. Yang, H. J. Qi, R. Zhao, *Adv. Mater.* **2021**, *33*, 2102113.
